# Limits and benefits of unsupervised transfer learning for Raman spectroscopy-based bioprocess monitoring

**DOI:** 10.1007/s00449-026-03394-8

**Published:** 2026-07-28

**Authors:** Bianca Hüpfl, Alexandra Umprecht, Bence Kozma, Peter Ettinger, Oliver Spadiut

**Affiliations:** 1 Institute of Chemical, Environmental and Bioscience Engineering, TU Wien, Gumpendorferstraße 1a, 1060 Vienna, Austria; 2 Baxalta Innovations GmbH, A Member of the Takeda Group of Companies, Industriestraße 67, 1220 Vienna, Austria; 3 Novartis Pharmaceutical Manufacturing GmbH, Biochemiestrasse 10, 6250 Kundl, Austria

**Keywords:** Raman spectroscopy, PAT, Bioprocess modeling, Unsupervised transfer learning, Domain adaptation, Preprocessing, PLS

## Abstract

Reliable analyte prediction from Raman spectra is critical for bioprocess monitoring, yet spectral variability across domains, such as different media or scale-up conditions, challenges model generalization. Models for analyte prediction are typically trained solely on labeled source-domain data, often limiting their performance under domain shifts. Transfer learning has been successful in other fields, but is underexplored for spectroscopic data in upstream bioprocesses. To help bridge this gap we assess whether unsupervised transfer learning methods can enhance model generalization by leveraging unlabeled target-domain spectra. We benchmark four unsupervised transfer learning approaches (CORAL, JDOT, TCA, OPP-MMD) against two bioprocess-relevant baselines: a fixed standard preprocessing workflow (REF) and an optimized source calibration based on systematic preprocessing screening (OPP). All methods are evaluated on two bioprocess Raman datasets: Dataset A comprises five glucose-spiked media and is complemented by in-silico spectral perturbations designed to mimic commonly occurring Raman noise types, such as baseline shifts, scattering artifacts, and spectral shifts. Dataset B evaluates scale-up transfer from pilot to production bioreactors for predicting glucose, biomass, and phosphate. Across the investigated cases, a consistent pattern emerged: JDOT and CORAL were most beneficial when across-domain differences dominated, whereas under high within-domain variability transfer learning provided no benefit and even underperformed the baseline. Because these transfer learning approaches can be integrated with standard Raman preprocessing pipelines, unsupervised transfer learning is best viewed as a complementary PAT tool rather than a universal replacement.

**Supplementary Information:**

The online version contains supplementary material available at https://doi.org/10.1007/s00449-026-03394-8.

## Introduction

The Process Analytical Technology (PAT) initiative has advanced bioprocess monitoring and control by integrating process sensors, modern computational resources, and chemometric modeling techniques [1–3]. Central to PAT is the real-time, non-invasive measurement of critical process parameters and critical quality attributes using spectroscopic methods such as near-infrared and Raman spectroscopy [4, 5]. Combined with multivariate modeling approaches such as partial least squares regression (PLS), these measurements can support real-time monitoring and control [6–8]. Among chemometric methods, PLS regression is widely used for spectral data modeling due to its ability to handle high-dimensional and collinear data typical for spectroscopy [9, 10]. PLS constructs a latent space by projecting spectral measurements (also termed predictors or features) onto components that maximize their covariance with the analyte concentration (also called response or labels), allowing modeling even when the number of spectral variables exceeds the number of samples. A key assumption behind PLS (and many other models) is that the spectra used to build the model and the spectra encountered later in routine use come from the same underlying distribution [11]. In this context, the source domain refers to the setting where the model is developed and calibrated (the available training spectra with known analyte concentrations), while the target domain refers to the new setting where the same model is later applied for prediction (new spectra, typically collected under different conditions). In practice, this assumption is frequently violated: spectra from shake flasks can differ systematically from spectra collected in larger reactors, and additional shifts can arise from changes in medium composition, equipment, or environmental conditions. As a result, models calibrated in one setting can show degraded predictive performance when applied in another [12–14]. Several factors contribute to this mismatch. In practice, spectra reflect more than analyte information: scattering effects, baseline variability, and measurement noise can mask or distort analyte-related signals [15]. If these contributions dominate, PLS may emphasize condition-specific artifacts rather than concentration-related variation, reducing model performance under changing measurement contexts. Spectral preprocessing is therefore routinely applied to suppress artifacts and to highlight analyte-related features. Since many preprocessing techniques and parameter settings exist, selection is still often performed by trial and error [9, 15, 16]. While this workflow is convenient, it can yield models that appear strong in the source domain but generalize poorly to new target spectra. To mitigate such effects, practitioners use sophisticated preprocessing techniques (including automated selection, ensembles, or orthogonal signal correction) [17–19] and established calibration-transfer workflows such as standardization, global models, or slope-and-bias correction [20, 21]. More recently, hybrid workflows combining classical calibration-transfer methods with mechanistic modeling have also been investigated [22]. However, these strategies can be insufficient when the source and target settings differ substantially, resulting in poor model performance even when careful preprocessing is applied [23, 24].

Transfer learning (TL) offers an alternative by incorporating information from both source and target domains to improve predictions under target conditions [25–27]. In the unsupervised setting considered here, only unlabeled target spectra are used during model training, which is an attractive scenario for bioprocesses, where obtaining reference measurements from new setups can be labor-intensive and expensive. TL has been applied successfully in several fields, including geology, agriculture, chemical engineering, forensics, medical diagnosis, and drug discovery [25, 28–34]. However, despite the prevalence of scale- and condition-induced spectral shifts in bioprocesses, only limited work has explored TL-guided preprocessing selection for Raman data in upstream use cases [35], and systematic comparisons to established TL methods are still missing. Accordingly, we evaluate whether unsupervised transfer learning can improve model performance of PLS-based models under bioprocess-relevant domain shifts, using only unlabeled target spectra. We benchmark four representative methods (CORAL, JDOT, TCA, and OPP-MMD) against two source-only baselines that reflect common PAT workflows: a fixed standard preprocessing chain and an optimized source calibration obtained by systematic preprocessing screening. The evaluation spans two upstream Raman datasets capturing pronounced domain variability in media composition and bioreactor scale. We further stress-test the methods by introducing controlled in-silico perturbations relevant to bioprocess Raman data (white noise, peak shifts, baseline drift) and label-distribution mismatch. Beyond reporting performance, we analyse when unsupervised TL improves upon the source-only baselines, suggesting that gains are analyte- and domain-dependent and can disappear under high within-domain variability. This positions TL as a complementary PAT tool alongside conventional workflows.

## Materials and methods

We evaluated all methods on two Raman datasets, restricting analysis to the 400–1800 cm$^{-1}$ region.

### Datasets

#### Dataset A (media transfer)

Dataset A includes spectra of five media (DMEM, F12, M9, LB and water) with a range of glucose spikes (Table 1). Mixtures of glucose and media were generated using the automated liquid handler of ambr®250 (Sartorius AG, Göttingen, Germany). A range of concentrated glucose stocks were prepared manually, their concentration verified by offline measurement using the Cedex®BioHT analyzer (Roche Custom Biotech, Penzberg, Germany) and added to a 24-deep well plate. 2.25 mL of each medium were dispensed into each well in a 48-deep-well plate, followed by the addition of 0.25 mL of glucose stock solution, resulting in 16 glucose concentrations between 0 and 20 g/L. Each medium was spiked with an identical set of glucose concentrations, and each medium–concentration combination was prepared in triplicate. Each mixture was mixed by pipetting and measured using the at-line Raman analyzer (HyperFlux^TM^ Raman Analyzer (Tornado, Mississauga, ON Canada)) integrated via the BioPAT®Spectra module (Sartorius AG, Göttingen, Germany). The Raman Analyzer is equipped with a 785 nm laser operating at a power of 495 mW. The spectral measurement range spans from $200~\textrm{cm}^{-1}$ to $3300~\textrm{cm}^{-1}$ (Raman shift) with a spectral resolution of $<5~\textrm{cm}^{-1}$. The acquisition settings were set to 1.2 s each and 30 accumulations were averaged for each sample. Acquisition settings were determined by adjusting settings based on medium with highest signal (TB, data not shown) that would result in 70% detector saturation. For each medium, the full sample set was prepared three times.

#### Dataset B (bioprocess scale-up)

Dataset B contains data from pilot (50 L and 150 L working volume) and production scale (14,000 L) main stage batches of a proprietary microbial (*Escherichia coli*) fed-batch fermentation for protein production (Table 1). The source and target domains therefore differ primarily in process scale, while the analyte ranges remain highly similar. The process is based on a chemically defined medium. During the main stage, carbon source and trace elements were continuously added. The process was operated under carbon-limited conditions, meaning that the carbon feed rate never exceeded the expected uptake by the microbial culture.

As a result, with the exception of the initial phase immediately after process initiation, glucose concentrations above $0.0~\textrm{g}/{L}$ should not occur. Transient glucose accumulation may occur under conditions of unintended overfeeding, for example when microbial metabolic activity declines. Accordingly, increasing glucose concentrations observed during the process may serve as an indicator of potential deviations from the intended process trajectory.

Raman spectra were collected inline using a Raman bIO-PRO probe (Kaiser Optical Systems, Ann Arbor, USA) installed in the bioreactor and connected to a Raman Rxn2 Analyzer (Endress+Hauser, Reinach, Switzerland). The analyzer was equipped with a 785 nm laser operated at 400 mW. Spectra were acquired over a Raman shift range of 100–$3425~\textrm{cm}^{-1}$ with a spectral resolution of $1~\textrm{cm}^{-1}$.

Each recorded spectrum was generated by averaging 60 consecutive individual acquisitions with an exposure time of 12.5 s each, resulting in a total integration time of 750 s per spectrum. Compared with the manufacturer-recommended setting of 10.0 s exposure time and 75 averaged acquisitions, the exposure time was increased to improve detector saturation, particularly during the early process phase. To keep the total integration time constant, the number of averaged acquisitions was reduced accordingly from 75 to 60. Spectral acquisition was performed periodically throughout the process, at least once per hour. Over the course of the process, detector saturation increased from approximately 10% to a maximum of 53%.

Offline analytics for reference samples was carried out with a SHIMADZU UV-1800 photometer for OD600 (Shimadzu, Kyoto, Japan) and with Thermo Scientific Arena 60 Analyzer for glucose and phosphate (Thermo Fisher, Massachusetts, USA). OD600 was used as a proxy for microbial biomass concentration.

**Table 1:** Overview of datasets and domains

Dataset	Use case	Source domain	Target domain	Analyte	$N_S$	$N_T$	$B_S$	$B_T$
A	Media transfer	DMEM, M9, F12, LB, or water	DMEM, M9, F12, LB, or water	Glucose	32	48 (if *S≠ T*); 16 (if *S=T*)	–	–
B	Scale transfer	Pilot scale	Production scale	Glucose, biomass, phosphate	1327	1427	15	22

$N_S,N_T$: number of spectra in source/target domain; $B_S,B_T$: number of batches in source/target domain

### Models

All modeling was performed using the following Python packages: chemotools [36], numpy [37], pandas [38], POT (Python Optimal Transport) [39], scikit-learn [40], pybaselines [41] and SKADA [42]. Six models were implemented, four of which leverage unlabeled target-domain spectra. For the models using target domain information and the reference model in Dataset A, standard normal variate (SNV) was consistently applied to the raw spectra as a preprocessing step. For the same models in Dataset B, data were preprocessed using a Savitzky–Golay first derivative filter followed by SNV. All model workflows are finalized with a Partial Least Squares (PLS) regression. The chosen model parameters can be found in Table S1 and Table S2 for Dataset A and B respectively.

*Partial least squares regression (PLS)* PLS is a regression technique that projects predictors and responses into a shared latent space to maximize their covariance, making it well-suited for high-dimensional, collinear data such as spectra [10, 43–45]. The built-in PLS implementation from scikit-learn was used, scanning 1–10 LVs. The optimal number of LVs was selected using a significance-based approach: Additional LVs were only included if they significantly reduced residuals (paired t-test, p = 0.05) [46]. To find the optimal number of LVs in CORAL, JDOT and TCA, a PLS model was trained on the source domain (5-fold CV; random splits). The found number of LVs was used for the PLS model after the domain adaptation step.

*Optimal Preprocessing (OPP)* The optimal preprocessing routine was based on the selection proposed by Engels et al. and is summarized in Table 2 [15]. For each preprocessing combination, a PLS model was trained on the source domain, and its performance was evaluated using 5-fold cross-validation (CV) with random splits. For all preprocessing combinations a significance test[46] was conducted to find the optimal number of latent variables (LVs) for a specific combination. The optimal model was chosen based on the lowest RMSE of cross-validation.

**Table 2:** Overview of applied preprocessing methods, grouped by correction type

Baseline	Scatter	Smoothing
None	None	Savitzky-Golay filter; no derivative and 1st order derivative window size: 10–60 (increments of 5) polynomial order: 2, 3, 4
1st–2nd order polynomial	Mean scaling	
AirPLS ($10^5$–$10^{10}$, multiplicative increase)	SNV	
AsLs (Asymmetric Least Squares) $\lambda : 10^4-10^7$; multiplicative increase *p* : 0.1, 0.01	Median scaling	
	Minmax scaling	
	Norm scaling	
	RNV	
	MSC	

*SNV* Standard normal variate, *RNV* Robust normal variate, *MSC* Multiplicative scatter correction, *AirPLS* Adaptive iteratively reweighted penalized least squares

*Base-case reference preprocessing (REF)* For Dataset A, REF is a PLS model that is built on SNV corrected data. In Dataset B, a Savitzky–Golay filter (window size 20, first order derivative, third order polynomial) was used in conjunction with SNV normalization to enhance spectral feature resolution. These represent examples of basic preprocessing steps that are used when no systematic screening of preprocessing combinations is conducted [15].

*Correlation Alignment (CORAL)* [47–50]

**Fig. 1:**
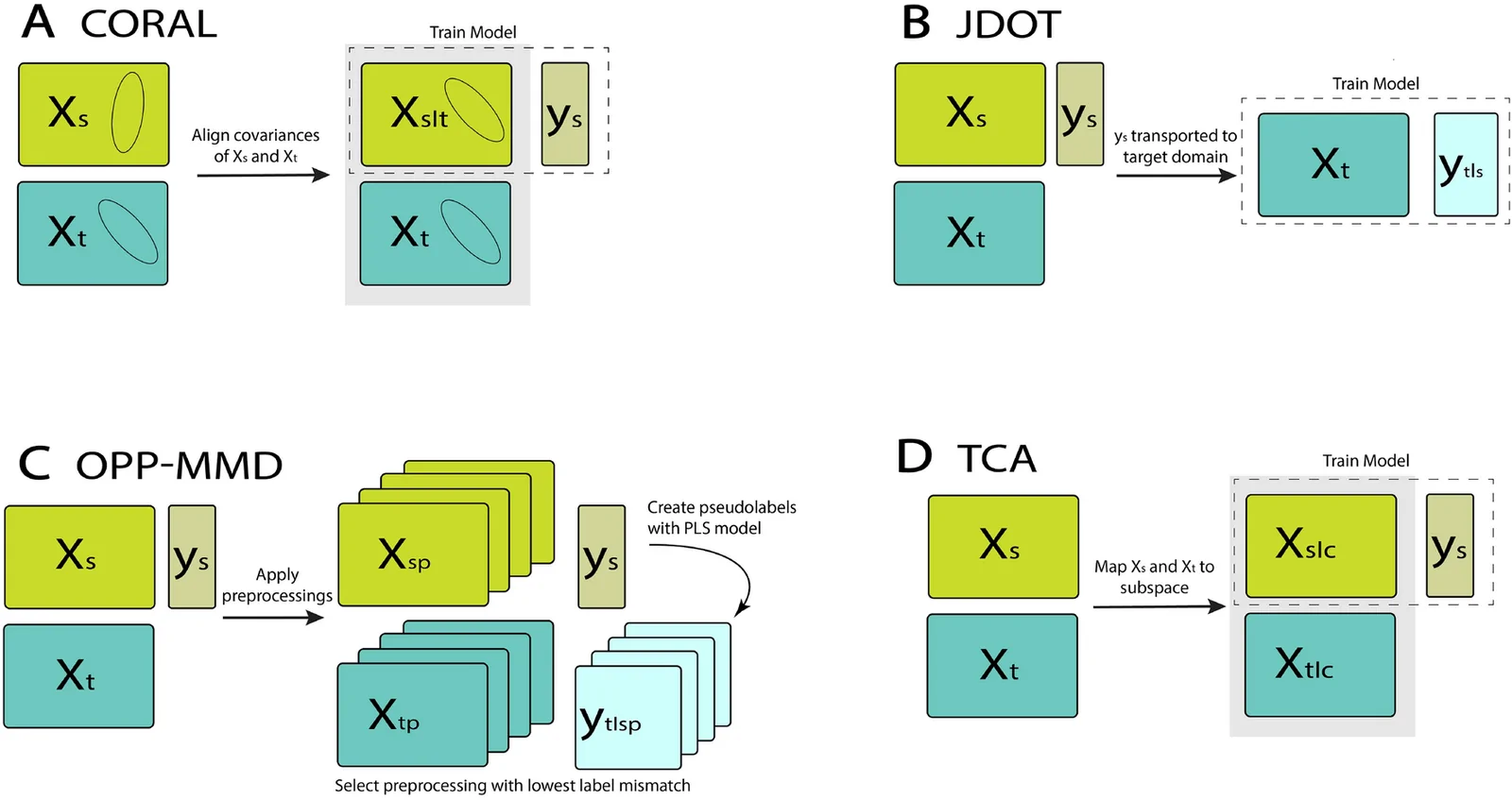
Graphic representation of model working mechanisms; CORAL = Correlation alignment, JDOT = Joint distribution optimal transport, OPP-MMD = Optimal preprocessing chosen by maximum mean discrepancy, TCA = Transfer component analysis; Xs = source domain spectra; Xt = target domain spectra; ys = source domain labels; Xs|t = Xs with aligned covariances with target domain; yt|s = target domain pseudo-labels created out of ys; Xsp/Xtp = Xs/Xt after preprocessing; yt|sp = preprocessed target domain pseudo-labels created out of ys; Xs|c/Xt|c = Xs/Xt mapped to shared component space; Xt|c = Xt mapped to subspace; grey background depicts a shared spectral domain; dashed rectangle depicts variables used for model training

CORAL is a domain adaptation method that aligns the second-order statistics (covariance matrices) of the source and target feature distributions (Fig. 1A). By minimizing the Frobenius norm between these covariances, CORAL transforms the source features to match the target distribution without requiring target labels. This simple, linear approach is computationally efficient and effective in reducing domain shift when variance structure is a key source of mismatch. In our workflow, Principal Component Analysis (PCA) was first applied to the combined source and target spectra, retaining components that explained 99% of the total variance. CORAL was then performed in this reduced space, followed by PLS regression on the aligned features.

*Joint Distribution Optimal Transport (JDOT)* [51–53]

JDOT is a domain adaptation method that aligns both the feature and label distributions between source and target domains using optimal transport (Fig. 1B). It computes a coupling between source and target samples by minimizing a cost function that combines feature distances and label prediction errors, using pseudo-labels for the unlabeled target data. This joint alignment enables JDOT to transfer models more effectively when domain shifts affect both input features and label structures. The *α * parameter, which balances feature and label alignment, was selected as described by Courty et al. [51].

*Optimal Preprocessing chosen by Maximum Mean Discrepancy (OPP-MMD)*

This procedure was conducted based on Umprecht et al. [35]. In our implementation, we first retained the top *5%* of preprocessing combinations according to the lowest RMSE on the source domain. For each corresponding PLS model, pseudo-labels were generated for the unlabeled target domain (Fig. 1C). The MMD between the source-domain labels and the target-domain pseudo-labels was then computed. The preprocessing combination yielding the lowest MMD was selected as the final optimal configuration. MMD is a statistical measure used to quantify domain differences. Here, it measures the domain distances between labels and pseudo-labels. A lower MMD indicates better alignment between domains, suggesting that the model generalizes more effectively to the target domain.

*Transfer Component Analysis (TCA)* [54–57]

TCA is a domain adaptation method that learns a shared feature space in which the marginal distributions of source and target domains are aligned (Fig. 1D). It achieves this by minimizing the Maximum Mean Discrepancy between domains while preserving the intrinsic structure of the data through kernel-based dimensionality reduction. Since TCA does not use label information, it is broadly applicable but may be less effective when task-relevant features differ across domains. TCA was applied to reduce the dimensionality of the spectra to 10 components and subsequently train a PLS model on this reduced space.

### Performance metrics

In this study, $R^2$, root mean squared error, and bias are used as complementary metrics to comprehensively evaluate model performance across domains. All used metrics are based on a comparison of the true values $y_i$ and the predicted values $\hat{y}_i$ for all samples *i=1,… ,n*. Furthermore, $\bar{y} = \frac{1}{n} \sum _{i=1}^n y_i$ refers to the average of the true values.

*Coefficient of determination *($R^2$) The Coefficient of determination

1 $$\begin{aligned} R^2 = 1 - \frac{\sum _{i=1}^{n} (y_i - \hat{y}_i)^2}{\sum _{i=1}^{n} (y_i - \bar{y})^2} \end{aligned}$$

quantifies the proportion of variance in the true analyte concentrations that is explained by the model, serving as a general indicator of goodness-of-fit.

*Normalized Root Mean Squared Error (nRMSE)*

In order to make the Root Mean Squared Error more comparable between domains and analytes with different scales we normalize it using the interquartile range (IQR) of the true values (*Q3 - Q1*), i.e.

2 $$\begin{aligned} \text {nRMSE} = \frac{\sqrt{\frac{1}{n} \sum _{i=1}^{n} (y_i - \hat{y}_i)^2}}{Q3 - Q1}. \end{aligned}$$

*where Q1 is the 25th percentile and Q3 is the 75th percentile of the dataset.*

*Normalized Bias (nBias)* In the same way as for the nRMSE, we normalize the bias

3 $$\begin{aligned} \text {nBias} = \frac{\frac{1}{n} \sum _{i=1}^{n} (y_i - \hat{y}_i)}{Q3 - Q1}. \end{aligned}$$

This gives us a scale-free measure of the magnitude of systematic prediction errors, revealing whether models consistently over- or under-predict.

*Maximum mean discrepancy (MMD)*

MMD was used to quantify distributional differences between spectral datasets. MMD estimates the distance between two probability distributions and is zero only when the two distributions are identical. In this study, MMD was applied both to quantify inter- and intra-domain variability. For the latter, each domain was split into two subsets based on the mean analyte concentration within that domain. Samples with analyte concentrations below the domain-specific mean were assigned to the “low” subset, whereas samples with concentrations above the mean were assigned to the “high” subset. The MMD between these two subsets was then used as a measure of intra-domain variability.

### Noise simulations

For Dataset A, common sources of distortion in Raman spectroscopy were simulated to assess their impact on transfer learning performance. Specifically, additive white noise, peak shifts, baseline drifts, and scattering were considered, that can arise from low signal-to-noise ratio, inter-instrument variability, fluorescence, and bubbles as well as biomass respectively [58–61]. In addition, the effect of label distribution mismatch was examined, a factor of particular concern for unsupervised approaches, as it can naturally occur due to differences in sampling strategies, experimental design, or biological variation between source and target domains [23].

*White Noise* We simulated in-domain noisy data by adding a flat white noise to all spectra with standard deviation 300 [a.u.]. For comparison, the root mean squares of the spectrum amplitudes after first-order baseline correction lie between 1200 [a.u.] and 1400 [a.u.].

*Peak Shifts* Spectral peak positions were perturbed by applying small random displacements along the wavenumber axis, representing calibration inconsistencies between instruments. After application of second-order baseline correction and smoothing, we receive the spectral data $\tilde{S}(\nu )$. We then warp the spectrum by applying a displacement field to the wavelengths, thereby warping the coordinates $\nu ^{\prime } = \nu + \alpha \tilde{S}(\nu )$, shifting the peaks in the spectrum, where we randomly choose a different *α * for each of the domains (see Table 3).

**Table 3:** Noise simulation parameters by domain

Domain	*α * (approx.)	$c^{\textrm{Bubble}}$	$c^{\textrm{Biomass}}$
LB	0.0017	5.0	1.2
F12	−0.0088	2.5	0.6
M9	−0.0030	0.0	0.0
DMEM	0.0137	5.0	0.6
${{\textrm{H}}_{2}{\textrm{O}}}$	−0.0198	0.0	0.6

*Baseline Drift*

To simulate complex between- and within-domain baseline shifts, we added a structured baseline *B(ν )* to each spectrum *X(ν )*, where *ν * denotes the Raman shift [cm$^{-1}$]. Each culture medium has a unique baseline shape with per-sample stochastic parameters. We write $\mathcal {N}(0,\sigma ^2)$ for a zero-mean Gaussian with standard deviation *σ * and use the logistic function $\textrm{sigmoid}(z)=\frac{1}{1+e^{-z}}$. Let $N_1\sim \mathcal {N}(0,\sigma _1^2)$ and $N_2\sim \mathcal {N}(0,\sigma _2^2)$ be sampled independently for each spectrum. The baselines are:

- *DMEM (linear downward tilt):*
  $$\begin{aligned} & B(\nu ) \;=\; N_1\,\nu + N_2,\quad N_1\sim \mathcal {N}(0,0.5^2),\\ & \quad N_2\sim \mathcal {N}(0,1000^2). \end{aligned}$$
- *M9 (linear upward tilt):*
  $$\begin{aligned} & B(\nu ) \;=\; (10 + N_1)\,\nu + N_2,\quad N_1\sim \mathcal {N}(0,0.5^2), \\ & \quad N_2\sim \mathcal {N}(0,1000^2). \end{aligned}$$
- *LB (central peak; single-cycle sine):*
  $$ B(\nu ) \;=\; \bigl (20000 + N_1\bigr )\,\sin \!\bigl (k\,\nu \,(1+N_2)\bigr ), $$
  where $N_1\sim \mathcal {N}(0,500^2)$, $N_2\sim \mathcal {N}(0,0.025^2)$, and *k* is chosen so that $\sin \!\bigl (k\,\nu \bigr )$ completes exactly one half-period over the spectral range, yielding a single peak and zeros at the range endpoints.
- *F12 (sigmoid-shaped hump):*
  $$ B(\nu ) \;=\; \bigl (20000 + N_1\bigr )\,\textrm{sigmoid}\!\bigl (k\,\nu \,(1+N_2)\bigr ), $$
  where $N_1\sim \mathcal {N}(0,500^2)$, $N_2\sim \mathcal {N}(0,0.05^2)$, and *k* is the same value as in the LB condition.
- *H*_*2*_*O* (inverted sigmoid dip):
  $$ B(\nu ) \;=\; \bigl (-10000 + N_1\bigr )\,\textrm{sigmoid}\!\bigl (k\,\nu \,(1+N_2)\bigr ), $$
  where $N_1\sim \mathcal {N}(0,500^2)$, $N_2\sim \mathcal {N}(0,0.05^2)$, and *k* matches the LB/F12 setting.

*Scattering artifacts* For simulating the effects of bubbles and biomass we build on the insights of Klaverdijk et al. [61]. We used AirPLS ($\lambda = 10^5$) to isolate a baseline and then multiplied the remaining part by medium-dependent mixtures of a bubble (constant $f^\textrm{Bubble}$) and biomass (non-constant $f^\textrm{Biomass}(\nu )$ spectral variation interpolated from values given in [61]) contributions

4 $$\begin{aligned} & \tilde{S}(\nu ) = B^\textrm{AirPLS}(\nu ) + (S(\nu )-B^\textrm{AirPLS}(\nu )) (1+ c^\textrm{Bubble} f^\textrm{Bubble}\nonumber \\ & \quad + c^\textrm{Biomass} f^\textrm{Biomass}(\nu ) ). \end{aligned}$$

The exact domain-specific contributions are summarized in Table 3.

*Label Distribution Mismatch* To induce uneven source–target label distributions, the source dataset was truncated by retaining only samples within a specified analyte range $[y_\text {min}, y_\text {max}]$. This procedure created systematic gaps in coverage relative to the target distribution.

## Results

The results are organized in three steps. First, Dataset A is used to evaluate model behavior under controlled medium-transfer conditions, where the glucose concentration range is matched across domains. Second, in-silico perturbations and MMD analyses are used to identify when transfer learning improves over source-only calibration. Third, Dataset B tests whether the same behavior is observed in an industrial pilot-to-production scale-transfer setting. Across both datasets, we distinguish between cases where inter-domain spectral differences dominate and cases where analyte-related spectral variation is sufficiently preserved across domains.

### Dataset A (media transfer)

**Fig. 2:**
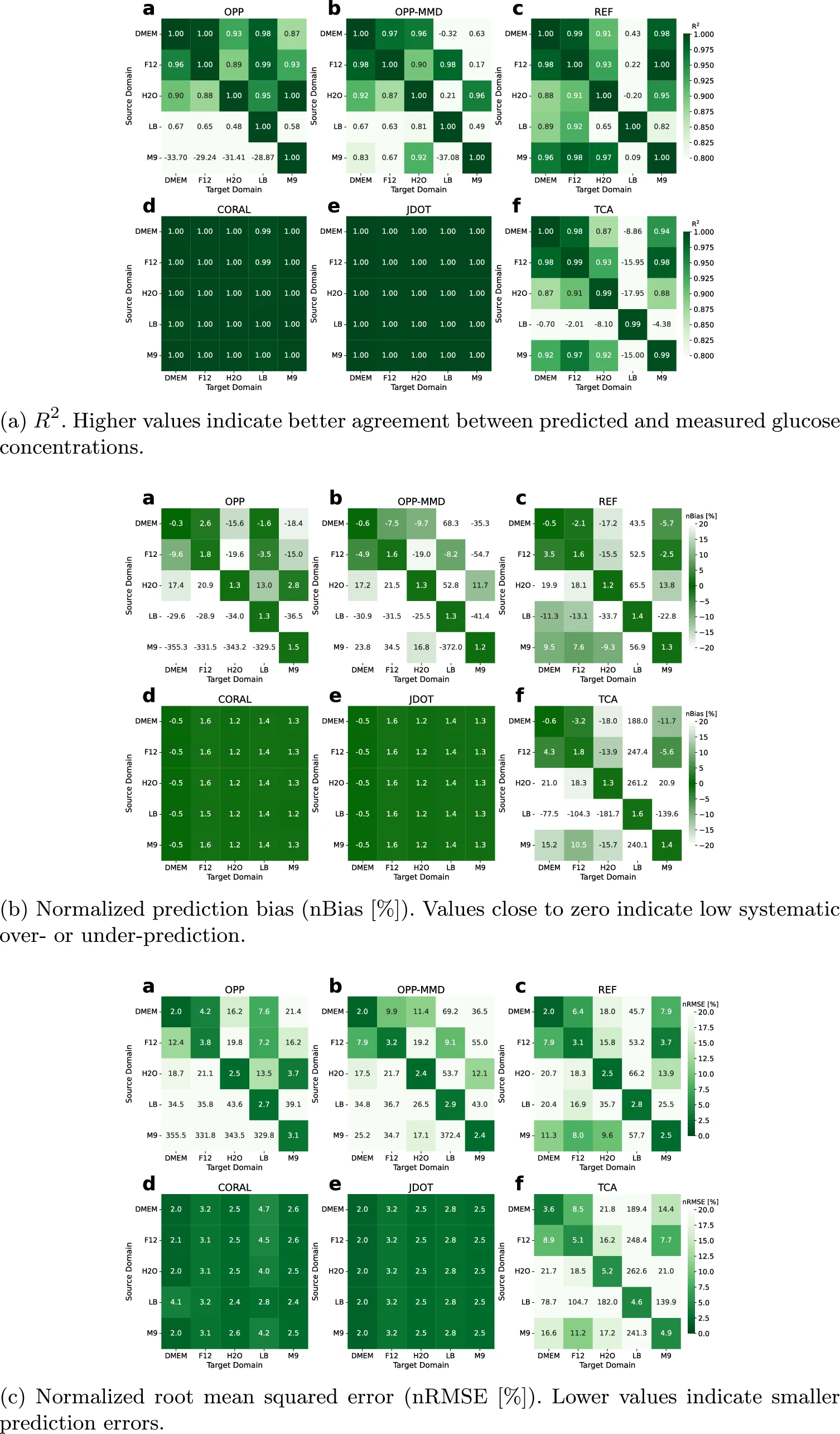
Model performance in Dataset A across multiple medium-to-medium transfer scenarios. Each subfigure compares six modeling approaches when trained on one medium and evaluated on the remaining media. **a** OPP = optimal preprocessing, **b** OPP-MMD = optimal preprocessing selected based on maximum mean discrepancy, **c** REF = reference preprocessing, **d** CORAL = correlation alignment, **e** JDOT = joint distribution optimal transport, and **f** TCA = transfer component analysis

Dataset A provides a controlled medium-transfer setting because all media were measured over the same glucose concentration range. Thus, differences in out-of-domain performance mainly reflect medium-induced spectral variation rather than differences in label coverage. Across all domain transfer scenarios, CORAL and JDOT consistently outperformed the other approaches in terms of predictive accuracy and generalization. Figure 2a shows that, for both methods, $R^2$ is equal to the in-domain training–testing baseline across all domain pairs (rounded to 1.00 across all domain pairs), indicating near-perfect agreement between predicted and true concentrations. Correspondingly, in Fig. 2b CORAL and JDOT achieved minimal systematic bias ($nBias\le |1.6| \%$) and Fig. 2c further confirms very low prediction error ($nRMSE \le 4.7 \%$). Overall, JDOT and CORAL achieved performance comparable to source-domain cross-validation in Dataset A (Fig. S1).

OPP, OPP-MMD, REF, and TCA yielded comparable performance metrics across most domain combinations; however, OPP and OPP-MMD performance varied markedly when M9 or LB were involved ($R^2$ between *-37.08* and 1.00, absolute bias between *372.0%* and *2.8%* and RMSE between *372.4 %* and *3.7 %*; ), whereas REF and TCA performance declined when LB was involved ($R^2$ between *-17.95* and 0.92, absolute bias between *261.2%* and *13.1%* and nRMSE between *262.6 %* and *16.9 %*; ).

**Fig. 3:**
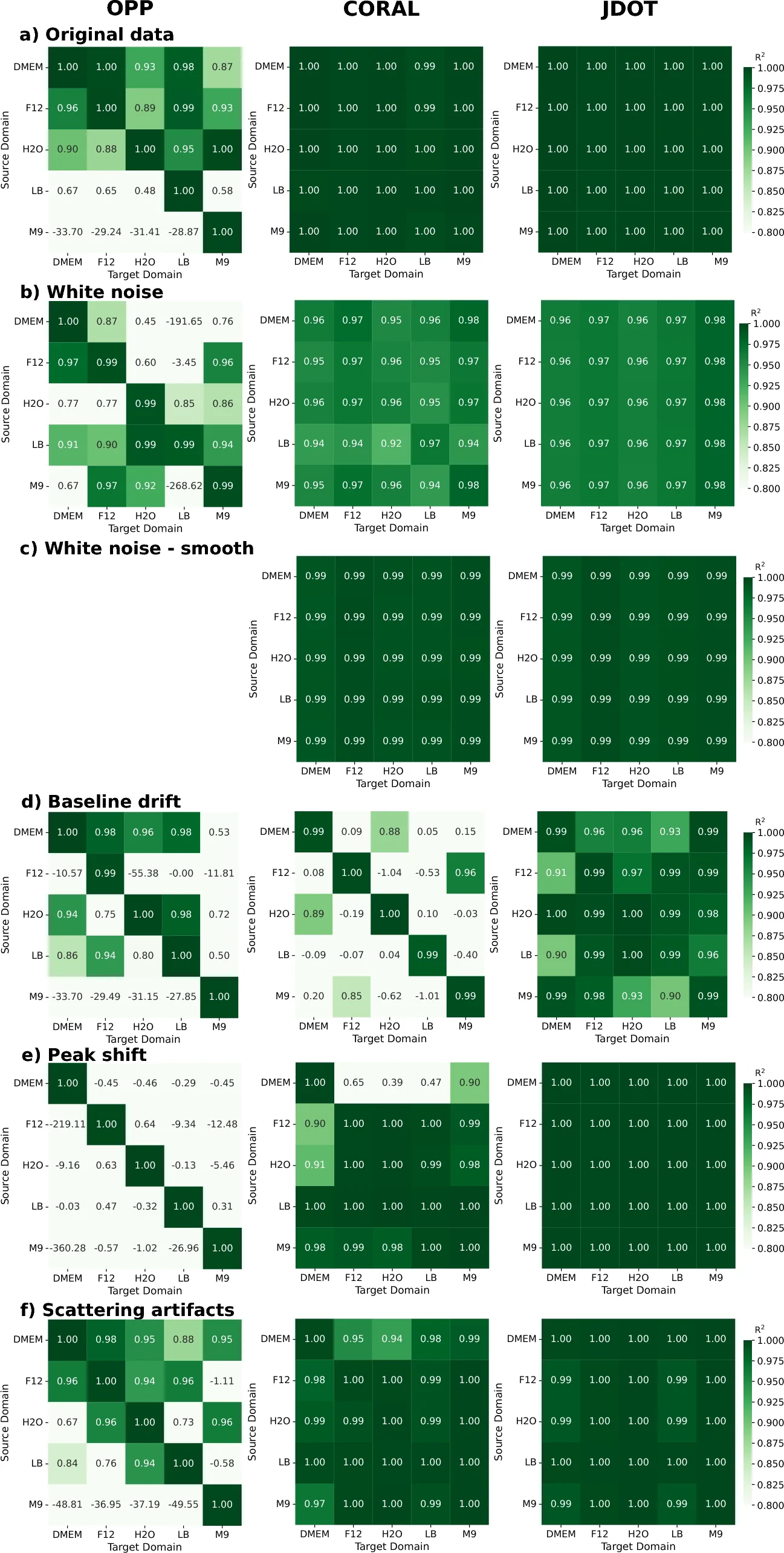
$R^2$ values for Dataset A across different domain-transfer scenarios and simulated Raman noise conditions. Columns show the three evaluated modeling approaches: OPP (optimized preprocessing), CORAL (correlation alignment), and JDOT (joint distribution optimal transport). Rows correspond to the individual noise scenarios (**a**–**f**). Each heatmap shows model performance when trained on one source domain and tested on the remaining target domains. Higher $R^2$ values indicate better agreement between predicted and measured glucose concentrations. The OPP model selected for **b** White noise and **c** White noise—smooth was identical; therefore, the corresponding heatmap is shown only once

To gain deeper insight into conditions underlying model success or failure, we introduced artificial distortions representative of common Raman artifacts: white noise, peak shifts, baseline drifts, and scattering artifacts, typically arising from low signal-to-noise ratio, inter-instrument variability, fluorescence, bubbles, and biomass, respectively [58–61]. In Fig. 3, $R^2$ values of the original data and all in-silico simulations of OPP, CORAL and JDOT are shown. Normalized RMSE and normalized bias showed comparable qualitative trends to the original, unperturbed data. The results of the remaining models are provided in Figs. S2 to S5.

Under the influence of white noise (Fig. 3b), OPP performance improved in some transfer scenarios and decreased in others. Initially JDOT and CORAL performed worse in this setting due to a degradation of the base model, but when combined with a smoothing step, both TL methods recovered to levels almost that of the domains without the added noise (Fig. 3c). Baseline distortions of varying intensity and shape (linear, sigmoid, sinusoidal) highlighted JDOT as the most robust and consistent approach (Fig. 3d). OPP occasionally matched its accuracy but exhibited substantial variability across domain combinations. When spectral peaks were shifted, JDOT again outperformed all other methods (Fig. 3e), followed by CORAL, while the other models failed to generalize outside a given source domain. The simulation of scattering artifacts showed a trend consistent with the other evaluated noise types, with JDOT performing best, followed by CORAL (Fig. 3f). Finally, we examined the impact of mismatched label distributions between source and target domains, a frequent challenge in unsupervised transfer caused by differences in sampling or biological variation [23]. Focusing on JDOT, the overall best-performing model, we examined transfer from DMEM to F12. In this scenario, JDOT’s performance declined under both mean and variance shifts (Fig. S10).

**Fig. 4:**
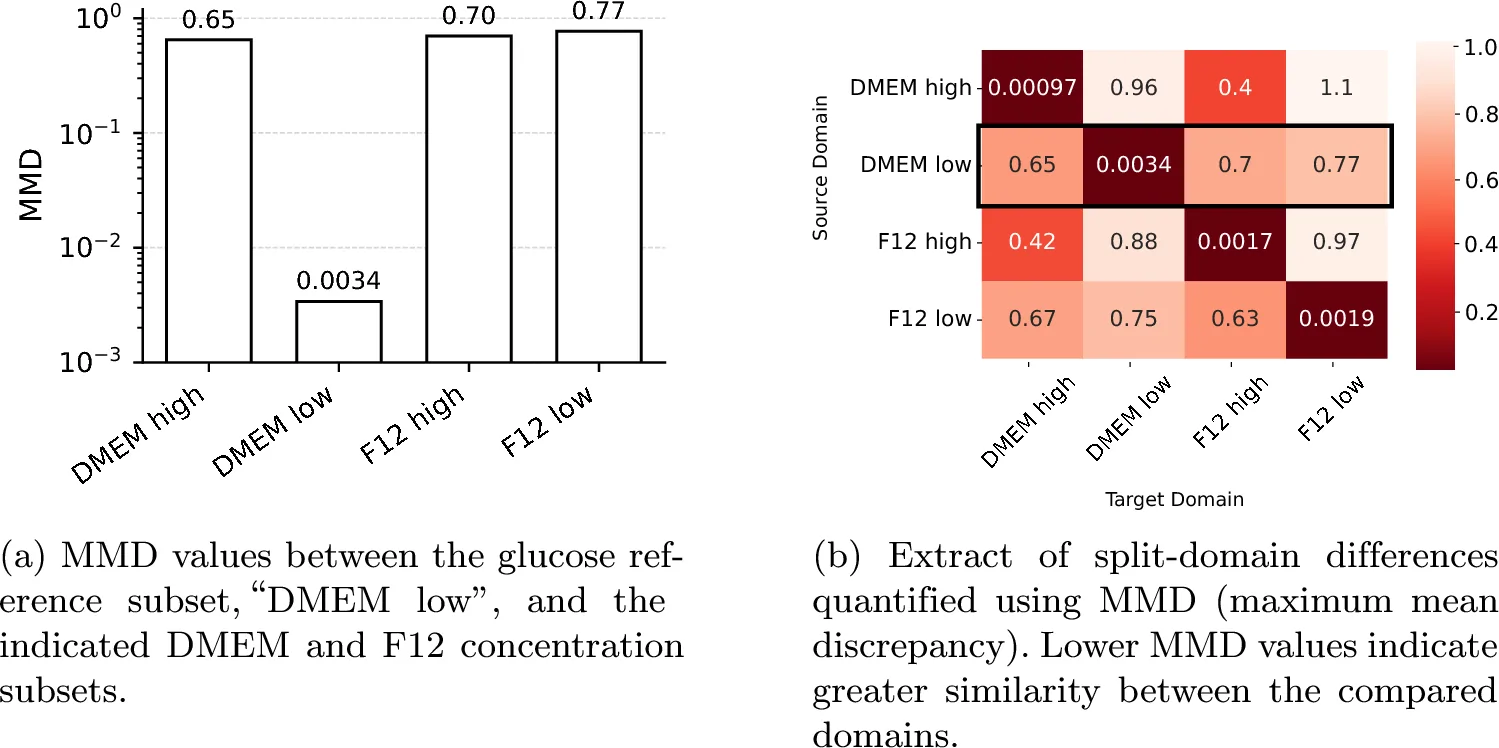
Examples of the MMD-based comparison of spectral subset variability in Dataset A

The MMD analysis was used to compare glucose-induced and medium-induced spectral variation. Spectra from each medium were split into low- and high-glucose subsets using the mean glucose concentration ($8.8~\mathrm {g/L}$). Within-medium low–high MMD values reflect glucose-related variation, whereas between-medium MMD values at comparable glucose levels reflect medium-related domain variation. If the former is much larger, source-only transfer is expected to be sufficient; if both are comparable, medium effects are relevant and transfer learning can provide an advantage. In Dataset A, within-medium and between-medium MMD values were of comparable magnitude, indicating that medium-induced variation was a relevant source of spectral mismatch. This is illustrated in Fig. 4a, where the MMD values from “DMEM low” to “DMEM high”, “F12 low”, and “F12 high” are similar. A subset of the corresponding MMD matrix is shown in Fig. 4b, and the complete matrix is provided in Fig. S12.

The regression coefficients of the evaluated models can be found in Fig. S13a and show distinct glucose-related Raman bands around $1125\,\textrm{cm}^{-1}$ and $517\,\textrm{cm}^{-1}$ [62]. For TCA, regression coefficients cannot be shown since this model is fitted after projection into a latent space.

### Dataset B (bioprocess scale-up)

**Fig. 5:**
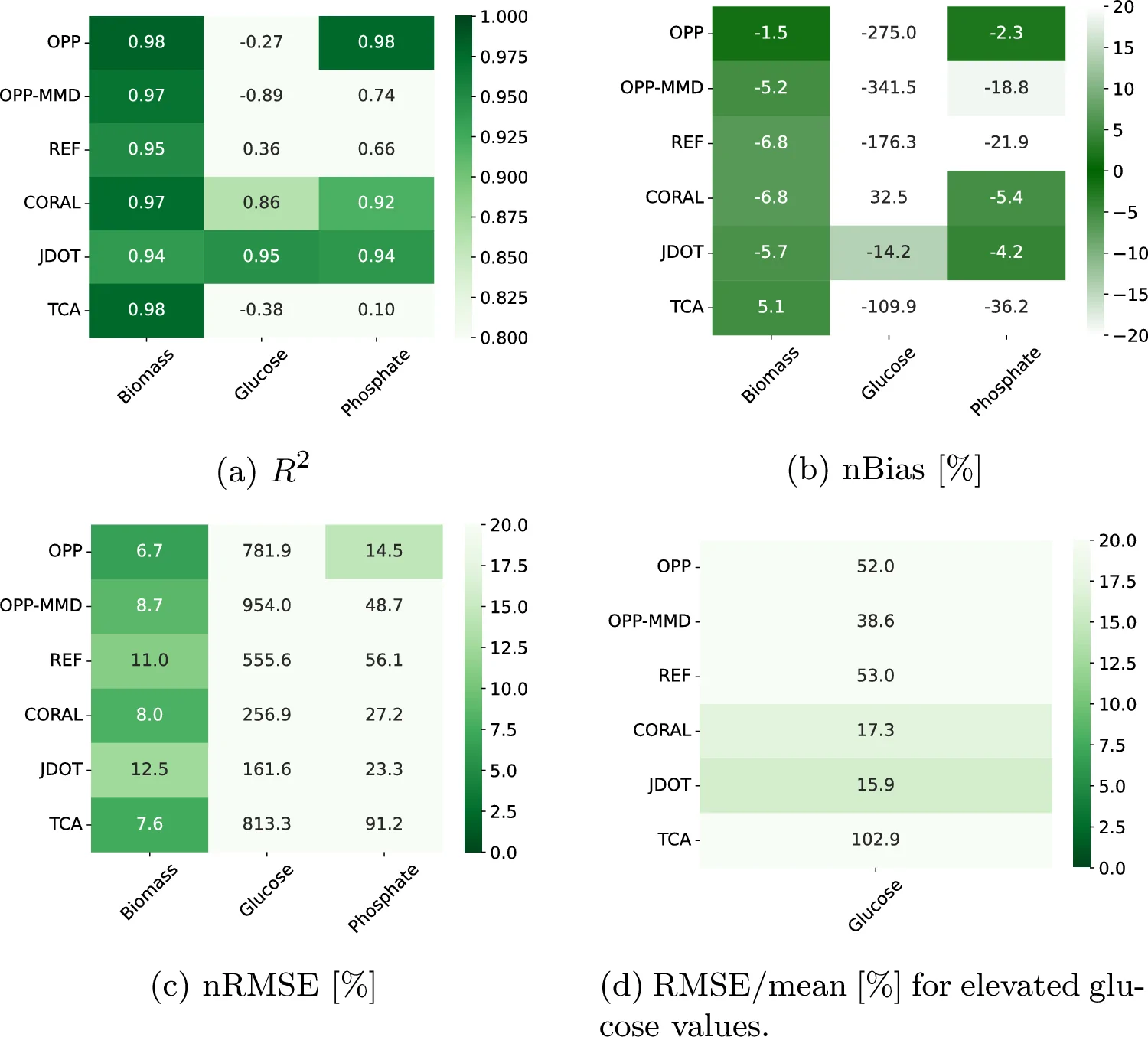
Performance metrics for Dataset B across six modeling techniques applied to a bioprocess scale-up scenario. Heatmap (**a**)–(**c**) summarizes one metric—$R^2$, nBias [%] or nRMSE [%]—across all analytes. $R^2$ = coefficient of determination, nBias [%] = normalized bias in percent, nRMSE [%] = normalized root mean squared error in percent, OPP = Optimal preprocessing, OPP-MMD = Optimal preprocessing chosen by maximum mean discrepancy, REF = Reference preprocessing, CORAL = Correlation alignment, JDOT = Joint distribution optimal transport, TCA = Transfer component analysis; each model workflow contains a preprocessing and/or transfer step followed by a PLS regression

Dataset B evaluated model transfer from pilot- to production-scale fermentations for three analytes: biomass, glucose, and phosphate. Model performance is summarized in Fig. 5 and differed between analytes.

For biomass, all evaluated models showed comparably strong performance with $R^2 \ge 0.94$, $|\textrm{nBias}| \le 6.8\%$, and $\textrm{nRMSE} \le 12.5\%$. OPP achieved the lowest prediction error for biomass, with $R^2 = 0.98$, $\textrm{nBias} = -1.5\%$, and $\textrm{nRMSE}=6.7\%$.

For glucose, the concentration distribution was strongly skewed, with many samples close to zero and fewer samples at elevated concentrations (Fig. S14). Therefore, global IQR-normalized errors were strongly influenced by the narrow interquartile range of the glucose distribution. Across all glucose samples, JDOT followed by CORAL achieved the lowest prediction error among the evaluated models, with $R^2 = 0.95$, $|\textrm{nBias}| = 14.2\%$, and $\textrm{nRMSE}=161.6\%$ for JDOT, and $R^2 = 0.86$, $|\textrm{nBias}| = 32.5\%$, and $\textrm{nRMSE}=256.9\%$ for CORAL. The source-only reference models showed larger errors and stronger prediction bias. Because the global glucose metrics were strongly affected by the narrow interquartile range of the skewed concentration distribution, prediction error was additionally calculated for the elevated-glucose samples only (Fig. 5d). In this subset, JDOT and CORAL showed RMSE values of *15.9%* and *17.3%* compared to the mean of the subset, respectively, whereas the source-only reference models OPP and REF showed RMSE values above *50%*.

For phosphate, OPP, JDOT, and CORAL showed the highest performance, with $R^2 \ge 0.92$, $|\textrm{nBias}| \le 5.4\%$, and $\textrm{nRMSE} \le 27.2\%$. Across analytes, JDOT showed consistently high model performance, with $R^2$ values above *0.90* for biomass, glucose, and phosphate. TCA and OPP-MMD did not improve over the source-only reference models. The regression coefficients for the analytes and models used on Dataset B are shown in Figs. S13b to S13d.

**Fig. 6:**
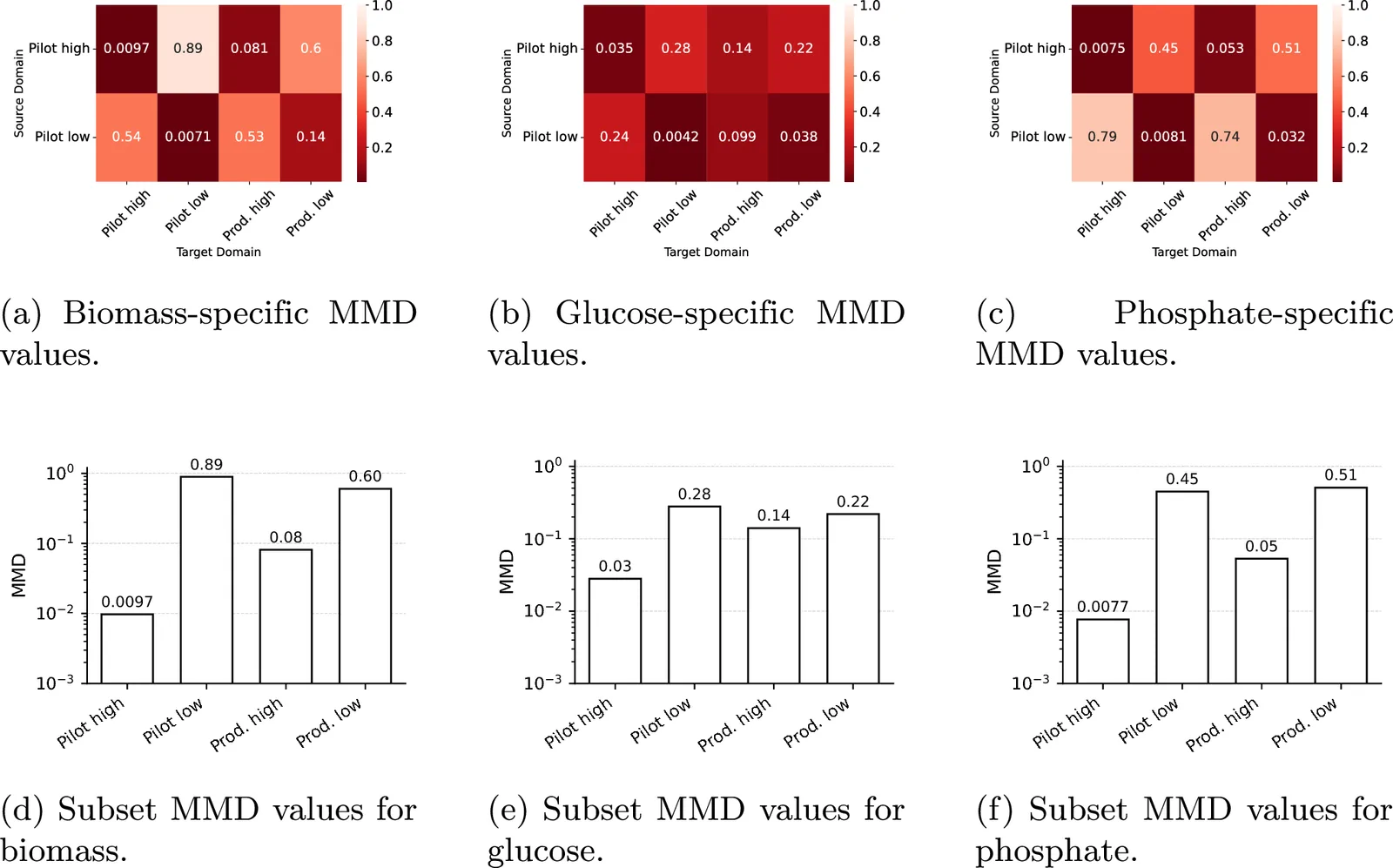
MMD-based analysis of spectral variability in Dataset B. Top row: analyte-specific MMD comparisons across pilot- and production-scale spectra for biomass, glucose, and phosphate. Lower MMD values indicate greater similarity between the compared spectral distributions. Bottom row: MMD values between the respective reference subset and the indicated pilot- or production-scale concentration subsets. For all analytes, the reference subset was “Pilot high”

The MMD analysis indicated analyte-dependent spectral variability patterns in the pilot-to-production transfer setting (Fig. 6).

## Discussion

This study set out to address the question of whether unsupervised transfer learning can enhance out-of-domain model performance in settings relevant for upstream bioprocessing. Such situations commonly occur, for example when a model calibrated in one cultivation medium is used in another medium, or when a model developed at small scale is transferred to pilot or production scale. We therefore evaluated transfer learning methods on both controlled spiked-media datasets and industrially relevant scale-up datasets. Overall, the results show that transfer learning did not universally surpass the benchmarks. However, selected transfer learning methods improved prediction performance when the spectral differences between source and target domains were larger than the variation within each domain condition.

Dataset A provided a well-controlled testbed: glucose was the analyte of interest, and different domains were created by switching between growth media. The same glucose distribution was present in all domains, such that the transfer task consisted of correcting medium-induced spectral differences. This dataset structure enabled a systematic comparison of model behavior under domain variability. The results show that JDOT and, except for simulated baseline shifts, also CORAL consistently outperformed the baseline model OPP, which was built on source-domain data alone (Fig. 3).

The MMD analysis helps to explain why these transfer learning approaches were beneficial in Dataset A. The comparison between low- and high-glucose groups in Fig. 4 showed that varying glucose concentrations as well as domain switches to another medium caused clear spectral changes. This effect is depicted in Fig. 4a: The MMD values between “DMEM low” and the other domain subgroups, “DMEM high”, “F12 high” and “F12 low”, are of comparable magnitude with 0.65, 0.70 and 0.77 respectively. This indicates that changing the medium introduced spectral variation that was similarly strong as the variation caused by glucose concentration within one medium. This observation is important for the interpretation of the model results. A model trained only in one medium may not only learn glucose-related spectral features, but also medium-specific spectral structures. When this model is then applied to another medium, these medium-related differences can reduce prediction performance. This provides a likely explanation for the weaker performance of the source-domain-based reference models OPP and REF in Dataset A. JDOT and CORAL are designed to reduce such differences between source and target spectra. CORAL aligns the covariance structure of the source and target data, while JDOT aligns samples by considering both spectral similarity and predicted label structure. Therefore, both methods can reduce medium-related spectral mismatch. The MMD results support this interpretation, because they show that medium effects were a relevant source of spectral variation in Dataset A.

To verify that TL methods perform beyond idealized settings, we introduced additional perturbations and noise types representative of Raman measurements. Overall, JDOT showed the best performance, and the gap to source-domain-based models (OPP, REF) tended to widen under perturbations. This is most apparent under peak shifts, which are common Raman artifacts arising among others from instrument-to-instrument variability [58]. JDOT largely mitigated systematic spectral misalignment, reaching accuracy close to a fully supervised PLS model trained and tested in the target domain (Fig. 3e). Its behavior is consistent with its optimal-transport alignment in joint feature–label space, which can mitigate moderate distribution shifts. CORAL also performed strongly under peak shifts, underscoring the effectiveness of fast, covariance-alignment approaches. In contrast, source-domain-based models deteriorated markedly, lacking a mechanism to handle shifted peaks.

Scattering artifacts, a common bioreactor effect caused by factors such as bubbles and biomass, were handled most effectively by JDOT and, for most domain combinations, also by CORAL (Fig. 3f). Under baseline drift across and within domains, JDOT showed the strongest performance and clearly exceeded all other methods, consistent with its label transport strategy (Fig. 3d). Overall, these results indicate that among the tested methods, JDOT is the least sensitive to several noise types commonly encountered in Raman spectroscopy for bioprocess monitoring.

When considering the presence of additional white noise, results highlight that preprocessing and TL (particularly CORAL and JDOT) act as complementary rather than competing components. Taking JDOT as an example, while JDOT manages to assign accurate pseudo-labels to the target domain data, the performance bottleneck is the base PLS model due to in-domain noise. By applying smoothing to the spectra, the PLS model performance can be improved, and near-optimal performance for JDOT and CORAL is regained (Fig. 3b and c).

We also observed a practical limitation of unsupervised transfer learning. When the glucose distribution in the target medium differed from the source medium (Fig. S10), JDOT performance decreased. Importantly, this was the only evaluated scenario in which the transfer task moved from interpolation toward extrapolation, because the target-domain analyte distribution was no longer fully covered by the source-domain calibration range. In line with literature, this indicates that unsupervised TL is sensitive to differences in analyte distributions between source and target domains [23]. Feature-based alignment methods such as CORAL and TCA face similar challenges, because they align spectral feature distributions but do not explicitly correct differences in the analyte distribution between source and target domains. Semi-supervised approaches, for example semi-supervised JDOT with a small number of labeled target samples, may reduce this limitation in practical applications.

If Dataset A showed the behavior of the transfer-learning methods under controlled medium shifts, Dataset B tested whether similar effects could also be observed in an industrially relevant bacterial bioprocess scale-transfer setting. The results were strongly analyte-dependent. For biomass and phosphate, where the MMD analysis indicated that analyte-related variation was pronounced (Fig. 6), the optimized PLS-based reference workflow performed competitively or better than the tested transfer-learning methods. This suggests that, when the analyte signal is sufficiently preserved across scales, careful preprocessing and PLS model optimization can already compensate for a substantial part of the domain difference.

Glucose showed a different behavior and has to be interpreted in the context of the glucose-limited process operation. After the initial process phase, glucose concentrations are expected to remain close to zero, whereas elevated glucose values indicate transient deviations from the intended carbon-limited state, for example due to overfeeding or reduced uptake. This process behavior is reflected in the strongly skewed glucose distribution in Dataset B, with many near-zero samples and only a smaller elevated-glucose subset (Fig. S14). As a result, global normalized error metrics are difficult to interpret for glucose, because the interquartile range is dominated by the near-zero concentration regime and small absolute errors can lead to large normalized errors. Therefore, the glucose results should not be interpreted as a broad quantitative calibration over a uniformly sampled concentration range, but in the context of a glucose-limited process with a dominant near-zero state and a smaller elevated-glucose subset.

For this process, the more relevant monitoring task is to distinguish glucose-depleted from elevated-glucose states and to recover the elevated-glucose level more accurately. In this elevated-glucose subset, JDOT and CORAL achieved lower errors than the source-only workflows, with RMSE values of *15.9%* and *17.3%* of the mean value, respectively, whereas OPP and REF showed errors above *50%* (Fig. 5d). This indicates that the transfer-learning methods predicted the elevated-glucose subset closer to the correct concentration level on average and improved the prediction behavior in the process-relevant glucose states.

**Fig. 7:**
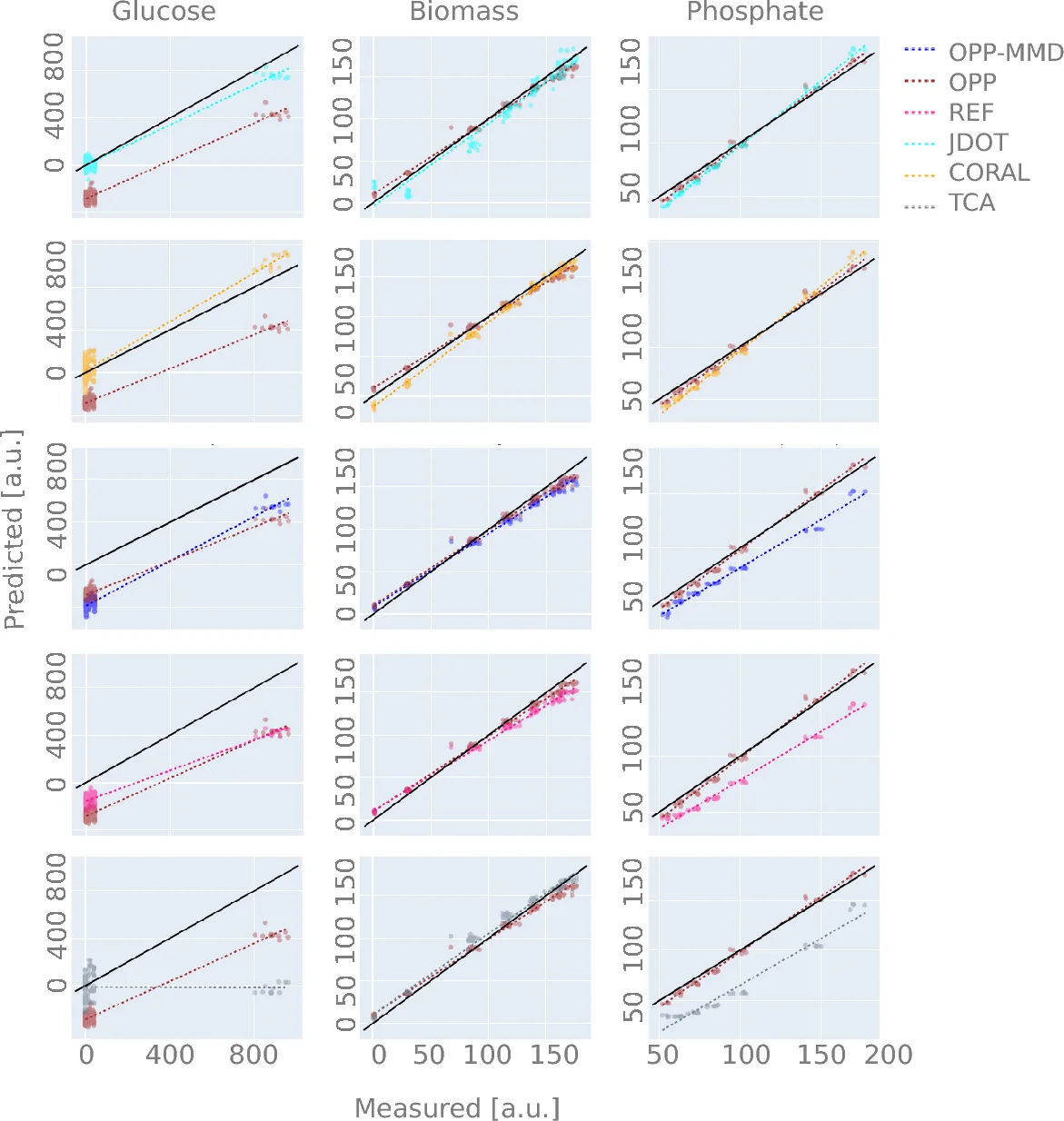
Measured-vs-predicted plots of analytes modeled in Dataset B. Data points were normalized according to the mean of a given analyte range. OPP = Optimal preprocessing, MMD = Maximum mean discrepancy, REF = Reference preprocessing, CORAL = Correlation alignment, JDOT = Joint distribution optimal transport, TCA = Transfer component analysis; each model workflow contains a preprocessing and/or transfer step followed by a PLS regression

The improvements of the transfer-learning methods can be understood by considering the measured-versus-predicted plots. These plots suggest that the improved prediction behavior was largely related to a reduction of the scale-dependent prediction offset (Fig. 7). The source-only models showed negative intercept shifts after transfer from pilot to production scale, whereas JDOT and CORAL showed intercepts closer to zero. This is consistent with the transfer mechanisms of the two methods: CORAL aligns second-order spectral statistics between pilot- and production-scale spectra, whereas JDOT additionally considers the joint structure of spectral features and predicted labels during transport.

Overall, Dataset B is consistent with the interpretation derived from Dataset A: unsupervised transfer learning is most useful when inter-domain spectral variability is large relative to analyte-induced variation. The glucose case illustrates this behavior in a challenging, glucose-limited process, where JDOT and CORAL mainly improved scale-transfer behavior by reducing the prediction offset and recovering the elevated-glucose state more accurately than the source-only workflows.

Across both datasets, the comparison between REF and OPP also showed that preprocessing selection remained an important determinant of model performance. For biomass and phosphate in Dataset B, OPP performed competitively or better than the tested transfer-learning methods, indicating that systematic preprocessing selection and PLS model optimization can already compensate for a substantial part of the domain difference when analyte-related spectral information is sufficiently preserved across scales. This also shows that transfer learning should not be viewed as a replacement for careful chemometric model development, but as a complementary strategy for cases where preprocessing and source-domain calibration alone are insufficient.

Overall, among the tested transfer-learning methods, JDOT and CORAL showed the most consistent benefits. CORAL reduces domain differences by aligning second-order spectral statistics between source and target spectra, whereas JDOT uses optimal transport to align source and target samples while also considering the predicted label structure. This likely explains why both methods were most beneficial in cases where scale- or medium-related spectral variation was large relative to the analyte-induced variation. In contrast, TCA, which had previously shown strong performance on IR spectra, failed here, perhaps due to Raman- and domain-specific factors [30]. This underscores that TL methods do not generalize uniformly across spectral modalities and therefore require careful method selection, tuning, and validation before deployment in upstream bioprocess applications.

From an industrial perspective, the intended use case of the best-performing transfer-learning method is to reduce the recalibration effort when moving to a related target process, such as a new medium, scale, or reactor. In such a workflow, a calibration model developed on the source process is adapted using unlabeled target spectra acquired during early runs, after which the adapted model can be used for on-line prediction. In practice, deployment would still require standard PAT workflows, including instrument qualification, a modeling pipeline, predefined acceptance limits, and ongoing model maintenance.

In this context, JDOT stands out not just for its solid performance under common noise types encountered in Raman spectroscopy during bioprocesses but also for its efficiency and ease of use. It required under a minute to process all domain pairs in Dataset A, required no labeled target data, and could be implemented directly in Python without extensive tuning. In contrast, OPP-based workflows were computationally expensive, often exceeding 30 min per analyte due to preprocessing selection and MMD evaluation. Importantly, JDOT was consistently competitive with the baselines REF and OPP and superior when domain shifts dominated, making it a practically attractive candidate for improving generalization when labeled target data are not yet available.

Still, this study has limitations. We used only PLS regression, which may bias results toward linear models. Our preprocessing pipeline for the transfer-learning workflows was fixed and not further optimized per analyte, as we focused on non–expert-driven choices; however, expert knowledge and more flexible customization of preprocessing—especially in combination with JDOT and CORAL—could further enhance results. As seen in this study, preprocessing and transfer learning are complementary, and their combination may yield additional performance gains. Also, the performance of OPP-MMD could likely be improved by choosing absolute thresholds adjusted and optimized per analyte, instead of a relative, uniform threshold. Moreover, we excluded semi-supervised approaches, even though a small number of labeled target samples could, in theory, help methods like JDOT resolve label alignment ambiguity. These constraints could affect method rankings and will need to be addressed in follow-up work.

Taken together, the results indicate that unsupervised transfer learning, particularly JDOT and CORAL, can improve Raman spectroscopy-based model transfer when domain-related spectral variation limits source-only calibration. However, the benefit depends on the relation between analyte-induced spectral variation and inter-domain spectral differences. When analyte-related variation is sufficiently preserved across domains, optimized preprocessing and PLS modeling can remain competitive. When domain differences dominate, unsupervised transfer learning provides a useful additional strategy for improving scale- or medium-transfer performance without requiring labeled target-domain samples during model development.

## Conclusion

This work evaluated four unsupervised transfer learning methods—CORAL, JDOT, OPP-MMD, and TCA—for predicting analyte concentrations from Raman spectra under both standardized media (Dataset A) and industrial scale-up (Dataset B) conditions. Our results highlight that JDOT, and in several cases also CORAL, offers a practical complement to preprocessing-driven modeling approaches. It was competitive with or outperformed OPP in several transfer scenarios and does not require extensive expert-guided preprocessing selection, making it attractive for bioprocess modeling. However, the advantage of JDOT was conditional and depended on the relation between domain-induced spectral variation and analyte-induced spectral variation. When the analyte contribution to domain variability was large, non-transfer alternatives performed comparably or better, suggesting that the benefits of TL are context-specific. Furthermore, the study was limited to fixed preprocessing routines, excluded semi-supervised alternatives, and relied solely on PLS regression as the predictive model. These constraints may have influenced the observed model rankings and should be addressed in future work.

Overall, especially JDOT represents a promising and computationally efficient domain adaptation in upstream bioprocessing, but the superiority is not guaranteed. Therefore, future work should focus on systematically investigating the influence of semi-supervised models and non-linear regression models, particularly in industrial-scale bioprocesses.

## Supplementary Information

Below is the link to the electronic supplementary material.

Supplementary file 1 (pdf 746 KB)

## Data Availability

The data used in this study are confidential.
