# Supplementary material for: Limits and benefits of unsupervised transfer learning for Raman spectroscopy-based bioprocess monitoring
Source: Bioprocess Biosyst Eng. 2026 Jul 28;49(10):2583–99. doi: 10.1007/s00449-026-03394-8 (PMC13582229; doi:10.1007/s00449-026-03394-8)
Supplement: Supplementary file 1 [file 449_2026_3394_MOESM1_ESM.pdf]

# 10 Supplementary Material

**Table S1**: Model parameters selected in Dataset A.

| Source | Target | Test | Model | Baseline Correction | Smoothing | Scatter Correction | # LVs |
|---|---|---|---|---|---|---|---|
| DMEM | DMEM | DMEM | OPP-MMD | 2ndorder | o2w55d0 | minmax | 1 |
| DMEM | F12 | F12 | OPP-MMD | AirPls9 | o2w50d0 | RNV | 3 |
| DMEM | H2O | H2O | OPP-MMD | AirPls10 | o2w45d1 | minmax | 2 |
| DMEM | LB | LB | OPP-MMD | AirPls9 | o2w50d0 | RNV | 3 |
| DMEM | M9 | M9 | OPP-MMD | AirPls10 | o2w45d1 | minmax | 2 |
| F12 | DMEM | DMEM | OPP-MMD | 2ndorder | o2w55d1 | minmax | 2 |
| F12 | F12 | F12 | OPP-MMD | 2ndorder | none | MSC | 1 |
| F12 | H2O | H2O | OPP-MMD | 2ndorder | none | MSC | 1 |
| F12 | LB | LB | OPP-MMD | 2ndorder | o2w55d1 | minmax | 2 |
| F12 | M9 | M9 | OPP-MMD | 2ndorder | o2w55d1 | minmax | 2 |
| H2O | DMEM | DMEM | OPP-MMD | AirPls10 | o4w20d0 | median | 3 |
| H2O | F12 | F12 | OPP-MMD | 2ndorder | none | MSC | 1 |
| H2O | H2O | H2O | OPP-MMD | 2ndorder | none | MSC | 1 |
| H2O | LB | LB | OPP-MMD | AirPls10 | o4w20d0 | median | 3 |
| H2O | M9 | M9 | OPP-MMD | 2ndorder | none | MSC | 1 |
| LB | DMEM | DMEM | OPP-MMD | AirPls8 | o2w15d0 | median | 3 |
| LB | F12 | F12 | OPP-MMD | AirPls8 | o2w15d0 | median | 3 |
| LB | H2O | H2O | OPP-MMD | AirPls7 | o2w35d0 | SNV | 2 |
| LB | LB | LB | OPP-MMD | 2ndorder | none | minmax | 1 |
| LB | M9 | M9 | OPP-MMD | AirPls8 | o2w15d0 | median | 3 |
| M9 | DMEM | DMEM | OPP-MMD | AsLS l5p0.100 | none | minmax | 2 |
| M9 | F12 | F12 | OPP-MMD | AsLS l5p0.100 | none | minmax | 2 |
| M9 | H2O | H2O | OPP-MMD | AsLS l5p0.100 | none | minmax | 2 |
| M9 | LB | LB | OPP-MMD | 2ndorder | o3w50d1 | RNV | 3 |
| M9 | M9 | M9 | OPP-MMD | 2ndorder | none | MSC | 1 |
| LB | LB | LB | OPP | AirPls8 | o2w35d0 | median | 3 |
| LB | LB | F12 | OPP | AirPls8 | o2w35d0 | median | 3 |
| LB | LB | M9 | OPP | AirPls8 | o2w35d0 | median | 3 |
| LB | LB | DMEM | OPP | AirPls8 | o2w35d0 | median | 3 |
| LB | LB | H2O | OPP | AirPls8 | o2w35d0 | median | 3 |
| F12 | F12 | LB | OPP | AirPls7 | o4w55d0 | minmax | 2 |
| F12 | F12 | F12 | OPP | AirPls7 | o4w55d0 | minmax | 2 |
| F12 | F12 | M9 | OPP | AirPls7 | o4w55d0 | minmax | 2 |
| F12 | F12 | DMEM | OPP | AirPls7 | o4w55d0 | minmax | 2 |
| F12 | F12 | H2O | OPP | AirPls7 | o4w55d0 | minmax | 2 |
| M9 | M9 | LB | OPP | AsLS l7p0.100 | o2w55d1 | minmax | 3 |
| M9 | M9 | F12 | OPP | AsLS l7p0.100 | o2w55d1 | minmax | 3 |

| Source | Target | Test | Model | Baseline Correction | Smoothing | Scatter Correction | # LVs |
|---|---|---|---|---|---|---|---|
| M9 | M9 | M9 | OPP | AsLS l7p0.100 | o2w55d1 | minmax | 3 |
| M9 | M9 | DMEM | OPP | AsLS l7p0.100 | o2w55d1 | minmax | 3 |
| M9 | M9 | H2O | OPP | AsLS l7p0.100 | o2w55d1 | minmax | 3 |
| DMEM | DMEM | LB | OPP | AsLS l6p0.010 | o3w55d0 | RNV | 3 |
| DMEM | DMEM | F12 | OPP | AsLS l6p0.010 | o3w55d0 | RNV | 3 |
| DMEM | DMEM | M9 | OPP | AsLS l6p0.010 | o3w55d0 | RNV | 3 |
| DMEM | DMEM | DMEM | OPP | AsLS l6p0.010 | o3w55d0 | RNV | 3 |
| DMEM | DMEM | H2O | OPP | AsLS l6p0.010 | o3w55d0 | RNV | 3 |
| H2O | H2O | LB | OPP | AsLS l6p0.010 | o2w35d0 | minmax | 2 |
| H2O | H2O | F12 | OPP | AsLS l6p0.010 | o2w35d0 | minmax | 2 |
| H2O | H2O | M9 | OPP | AsLS l6p0.010 | o2w35d0 | minmax | 2 |
| H2O | H2O | DMEM | OPP | AsLS l6p0.010 | o2w35d0 | minmax | 2 |
| H2O | H2O | H2O | OPP | AsLS l6p0.010 | o2w35d0 | minmax | 2 |
| DMEM | DMEM | DMEM | SNV | none | none | SNV | 1 |
| DMEM | DMEM | F12 | SNV | none | none | SNV | 1 |
| DMEM | DMEM | H2O | SNV | none | none | SNV | 1 |
| DMEM | DMEM | LB | SNV | none | none | SNV | 1 |
| DMEM | DMEM | M9 | SNV | none | none | SNV | 1 |
| F12 | F12 | DMEM | SNV | none | none | SNV | 1 |
| F12 | F12 | F12 | SNV | none | none | SNV | 1 |
| F12 | F12 | H2O | SNV | none | none | SNV | 1 |
| F12 | F12 | LB | SNV | none | none | SNV | 1 |
| F12 | F12 | M9 | SNV | none | none | SNV | 1 |
| H2O | H2O | DMEM | SNV | none | none | SNV | 1 |
| H2O | H2O | F12 | SNV | none | none | SNV | 1 |
| H2O | H2O | H2O | SNV | none | none | SNV | 1 |
| H2O | H2O | LB | SNV | none | none | SNV | 1 |
| H2O | H2O | M9 | SNV | none | none | SNV | 1 |
| LB | LB | DMEM | SNV | none | none | SNV | 1 |
| LB | LB | F12 | SNV | none | none | SNV | 1 |
| LB | LB | H2O | SNV | none | none | SNV | 1 |
| LB | LB | LB | SNV | none | none | SNV | 1 |
| LB | LB | M9 | SNV | none | none | SNV | 1 |
| M9 | M9 | DMEM | SNV | none | none | SNV | 1 |
| M9 | M9 | F12 | SNV | none | none | SNV | 1 |
| M9 | M9 | H2O | SNV | none | none | SNV | 1 |
| M9 | M9 | LB | SNV | none | none | SNV | 1 |
| M9 | M9 | M9 | SNV | none | none | SNV | 1 |
| DMEM | DMEM | DMEM | JDOT | none | none | SNV | 1 |
| DMEM | F12 | F12 | JDOT | none | none | SNV | 1 |
| DMEM | H2O | H2O | JDOT | none | none | SNV | 1 |
| DMEM | LB | LB | JDOT | none | none | SNV | 1 |

| Source | Target | Test | Model | Baseline Correction | Smoothing | Scatter Correction | # LVs |
|---|---|---|---|---|---|---|---|
| DMEM | M9 | M9 | JDOT | none | none | SNV | 1 |
| F12 | DMEM | DMEM | JDOT | none | none | SNV | 1 |
| F12 | F12 | F12 | JDOT | none | none | SNV | 1 |
| F12 | H2O | H2O | JDOT | none | none | SNV | 1 |
| F12 | LB | LB | JDOT | none | none | SNV | 1 |
| F12 | M9 | M9 | JDOT | none | none | SNV | 1 |
| H2O | DMEM | DMEM | JDOT | none | none | SNV | 1 |
| H2O | F12 | F12 | JDOT | none | none | SNV | 1 |
| H2O | H2O | H2O | JDOT | none | none | SNV | 1 |
| H2O | LB | LB | JDOT | none | none | SNV | 1 |
| H2O | M9 | M9 | JDOT | none | none | SNV | 1 |
| LB | DMEM | DMEM | JDOT | none | none | SNV | 1 |
| LB | F12 | F12 | JDOT | none | none | SNV | 1 |
| LB | H2O | H2O | JDOT | none | none | SNV | 1 |
| LB | LB | LB | JDOT | none | none | SNV | 1 |
| LB | M9 | M9 | JDOT | none | none | SNV | 1 |
| M9 | DMEM | DMEM | JDOT | none | none | SNV | 1 |
| M9 | F12 | F12 | JDOT | none | none | SNV | 1 |
| M9 | H2O | H2O | JDOT | none | none | SNV | 1 |
| M9 | LB | LB | JDOT | none | none | SNV | 1 |
| M9 | M9 | M9 | JDOT | none | none | SNV | 1 |
| DMEM | DMEM | DMEM | CORAL | none | none | SNV | 1 |
| DMEM | F12 | F12 | CORAL | none | none | SNV | 1 |
| DMEM | H2O | H2O | CORAL | none | none | SNV | 1 |
| DMEM | LB | LB | CORAL | none | none | SNV | 1 |
| DMEM | M9 | M9 | CORAL | none | none | SNV | 1 |
| F12 | DMEM | DMEM | CORAL | none | none | SNV | 1 |
| F12 | F12 | F12 | CORAL | none | none | SNV | 1 |
| F12 | H2O | H2O | CORAL | none | none | SNV | 1 |
| F12 | LB | LB | CORAL | none | none | SNV | 1 |
| F12 | M9 | M9 | CORAL | none | none | SNV | 1 |
| H2O | DMEM | DMEM | CORAL | none | none | SNV | 1 |
| H2O | F12 | F12 | CORAL | none | none | SNV | 1 |
| H2O | H2O | H2O | CORAL | none | none | SNV | 1 |
| H2O | LB | LB | CORAL | none | none | SNV | 1 |
| H2O | M9 | M9 | CORAL | none | none | SNV | 1 |
| LB | DMEM | DMEM | CORAL | none | none | SNV | 1 |
| LB | F12 | F12 | CORAL | none | none | SNV | 1 |
| LB | H2O | H2O | CORAL | none | none | SNV | 1 |
| LB | LB | LB | CORAL | none | none | SNV | 1 |
| LB | M9 | M9 | CORAL | none | none | SNV | 1 |
| M9 | DMEM | DMEM | CORAL | none | none | SNV | 1 |

| Source | Target | Test | Model | Baseline Correction | Smoothing | Scatter Correction | # LVs |
|---|---|---|---|---|---|---|---|
| M9 | F12 | F12 | CORAL | none | none | SNV | 1 |
| M9 | H2O | H2O | CORAL | none | none | SNV | 1 |
| M9 | LB | LB | CORAL | none | none | SNV | 1 |
| M9 | M9 | M9 | CORAL | none | none | SNV | 1 |
| DMEM | DMEM | DMEM | TCA | none | none | SNV | 1 |
| DMEM | F12 | F12 | TCA | none | none | SNV | 1 |
| DMEM | H2O | H2O | TCA | none | none | SNV | 1 |
| DMEM | LB | LB | TCA | none | none | SNV | 1 |
| DMEM | M9 | M9 | TCA | none | none | SNV | 1 |
| F12 | DMEM | DMEM | TCA | none | none | SNV | 1 |
| F12 | F12 | F12 | TCA | none | none | SNV | 1 |
| F12 | H2O | H2O | TCA | none | none | SNV | 1 |
| F12 | LB | LB | TCA | none | none | SNV | 1 |
| F12 | M9 | M9 | TCA | none | none | SNV | 1 |
| H2O | DMEM | DMEM | TCA | none | none | SNV | 1 |
| H2O | F12 | F12 | TCA | none | none | SNV | 1 |
| H2O | H2O | H2O | TCA | none | none | SNV | 1 |
| H2O | LB | LB | TCA | none | none | SNV | 1 |
| H2O | M9 | M9 | TCA | none | none | SNV | 1 |
| LB | DMEM | DMEM | TCA | none | none | SNV | 1 |
| LB | F12 | F12 | TCA | none | none | SNV | 1 |
| LB | H2O | H2O | TCA | none | none | SNV | 1 |
| LB | LB | LB | TCA | none | none | SNV | 1 |
| LB | M9 | M9 | TCA | none | none | SNV | 1 |
| M9 | DMEM | DMEM | TCA | none | none | SNV | 1 |
| M9 | F12 | F12 | TCA | none | none | SNV | 1 |
| M9 | H2O | H2O | TCA | none | none | SNV | 1 |
| M9 | LB | LB | TCA | none | none | SNV | 1 |
| M9 | M9 | M9 | TCA | none | none | SNV | 1 |

**Table S2**: Model parameters selected in Dataset B.

| Source | Target | Test | Model | Baseline Correction | Smoothing | Scatter Correction | # LVs | Analyte |
|---|---|---|---|---|---|---|---|---|
| Pilot | Pilot | Prod. | OPP | AsLS l6p0.100 | o2w15d0 | RNV | 5 | Glucose |
| Pilot | Pilot | Prod. | OPP | AirPls5 | o4w40d1 | SNV | 3 | Biomass |
| Pilot | Pilot | Prod. | OPP | AsLS l7p0.010 | o2w25d1 | median | 3 | Phosphate |
| Pilot | Prod. | Prod. | OPP-MMD | 2ndorder | o2w15d1 | none | 6 | Glucose |
| Pilot | Prod. | Prod. | OPP-MMD | AirPls5 | o2w30d1 | minmax | 2 | Biomass |
| Pilot | Prod. | Prod. | OPP-MMD | 2ndorder | o2w35d1 | RNV | 2 | Phosphate |
| Pilot | Prod. | Prod. | JDOT | none | o3w20 | SNV | 5 | Glucose |
| Pilot | Prod. | Prod. | JDOT | none | o3w20 | SNV | 4 | Biomass |
| Pilot | Prod. | Prod. | JDOT | none | o3w20 | SNV | 1 | Phosphate |

| Source | Target | Test | Model | Baseline Correction | Smoothing | Scatter Correction | # LVs | Analyte |
|---|---|---|---|---|---|---|---|---|
| Pilot | Prod. | Prod. | CORAL | none | o3w20 | SNV | 5 | Glucose |
| Pilot | Prod. | Prod. | CORAL | none | o3w20 | SNV | 4 | Biomass |
| Pilot | Prod. | Prod. | CORAL | none | o3w20 | SNV | 1 | Phosphate |
| Pilot | Prod. | Prod. | TCA | none | o3w20 | SNV | 5 | Glucose |
| Pilot | Prod. | Prod. | TCA | none | o3w20 | SNV | 4 | Biomass |
| Pilot | Prod. | Prod. | TCA | none | o3w20 | SNV | 1 | Phosphate |

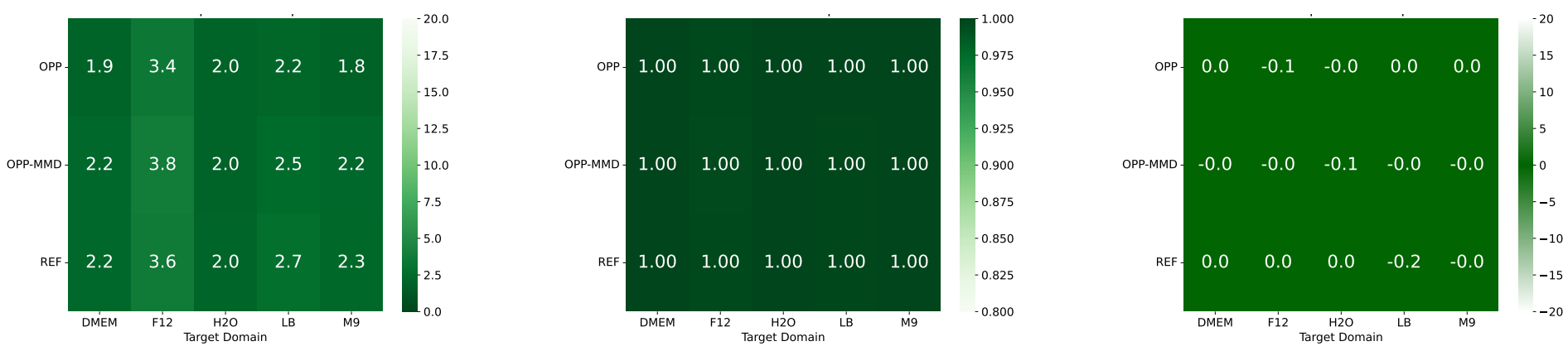


(a) RMSE [%] (b) $R^2$ (c) Bias [%]

**Fig. S1**: Performance metrics of source domain calculated by cross-validation (CV; 5-fold, random splits) on Dataset A. OPP = Optimal preprocessing, OPP-MMD = Optimal preprocessing chosen by maximum mean discrepancy, REF = Reference preprocessing

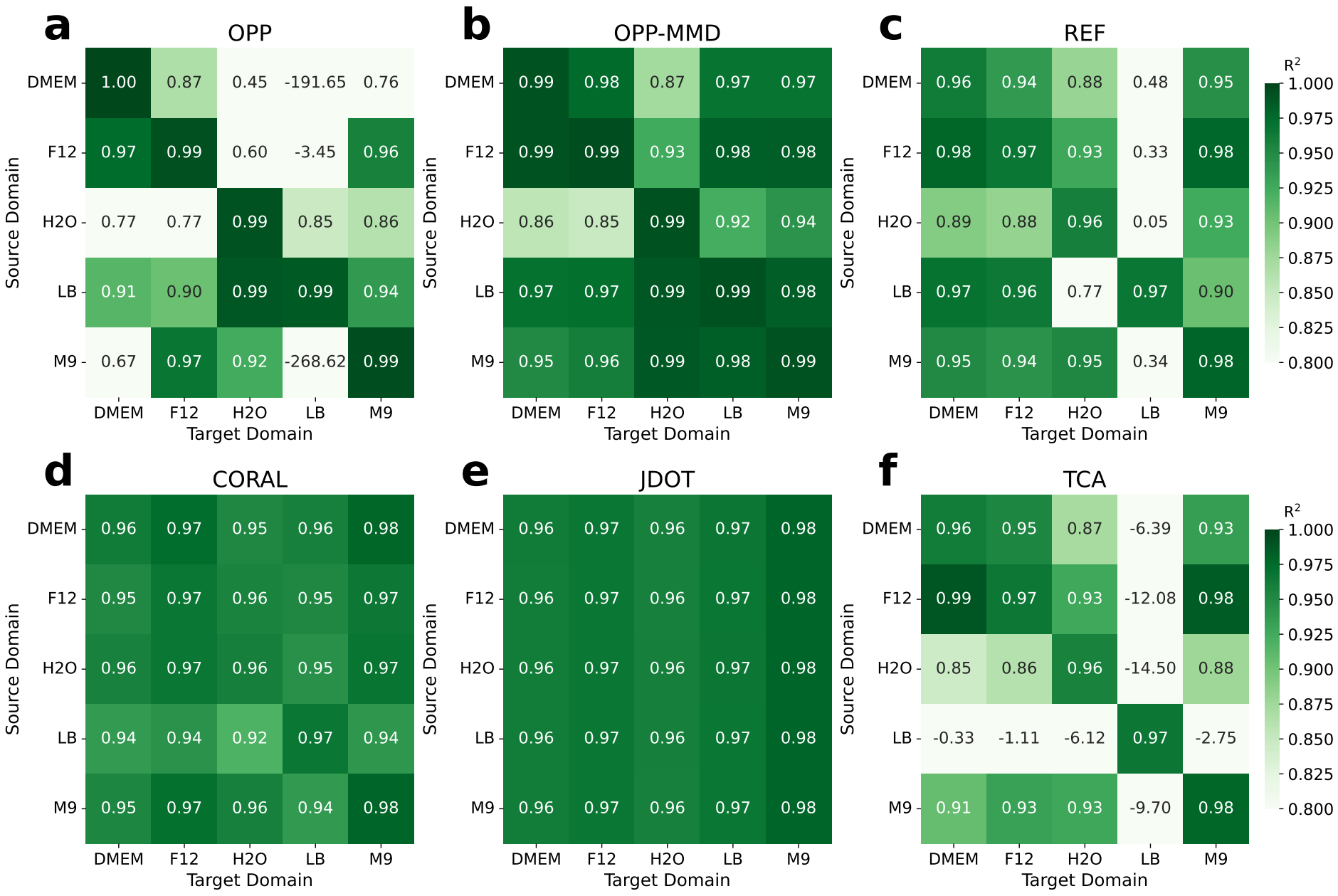


(a) White noise.

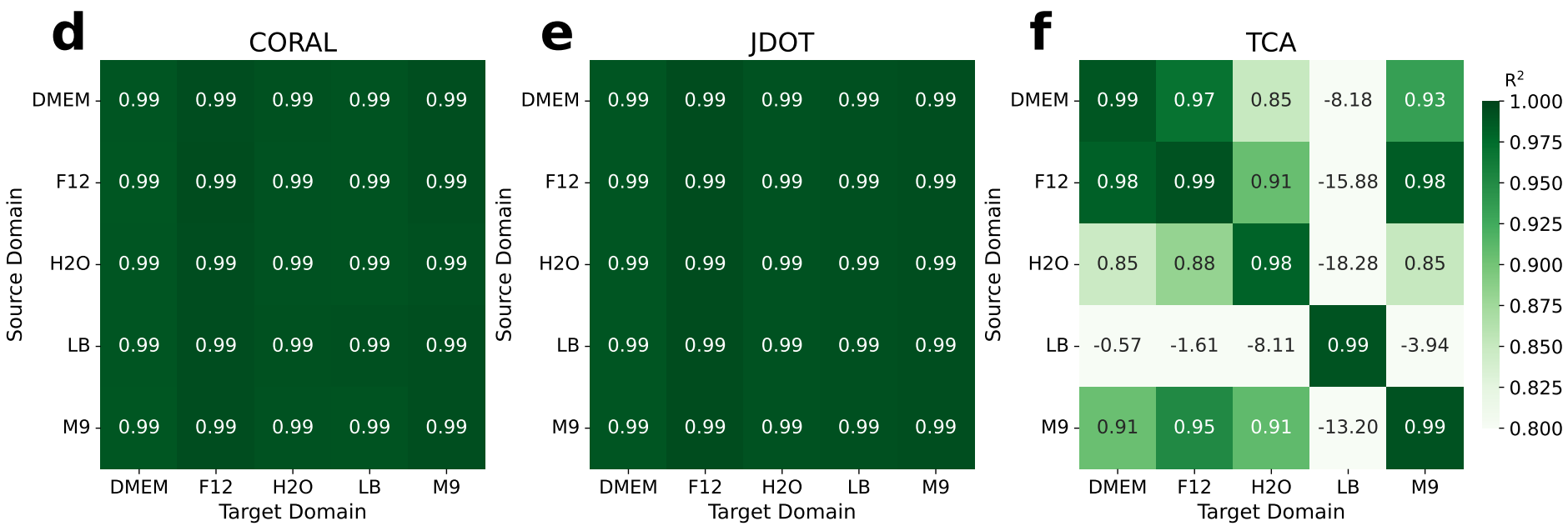


(b) White noise with smoothing step.

**Fig. S2**: Coefficient of determination ($R^2$) in Dataset A for six different modeling techniques under addition of white noise, shown without enforced smoothing in (a) and with an additional smoothing step in the preprocessing routine of CORAL, JDOT and TCA in (b). Each sub-plot corresponds to one model and shows performance when trained on one domain and tested on all others. Higher $R^2$ values indicate better agreement between predictions and true values. a) OPP = Optimal preprocessing, b) OPP-MMD = Optimal preprocessing chosen by maximum mean discrepancy, c) REF = Reference preprocessing, d) CORAL = Correlation alignment, e) JDOT = Joint distribution optimal transport, f) TCA = Transfer component analysis; each model workflow contains a preprocessing and/or transfer step followed by a PLS regression.

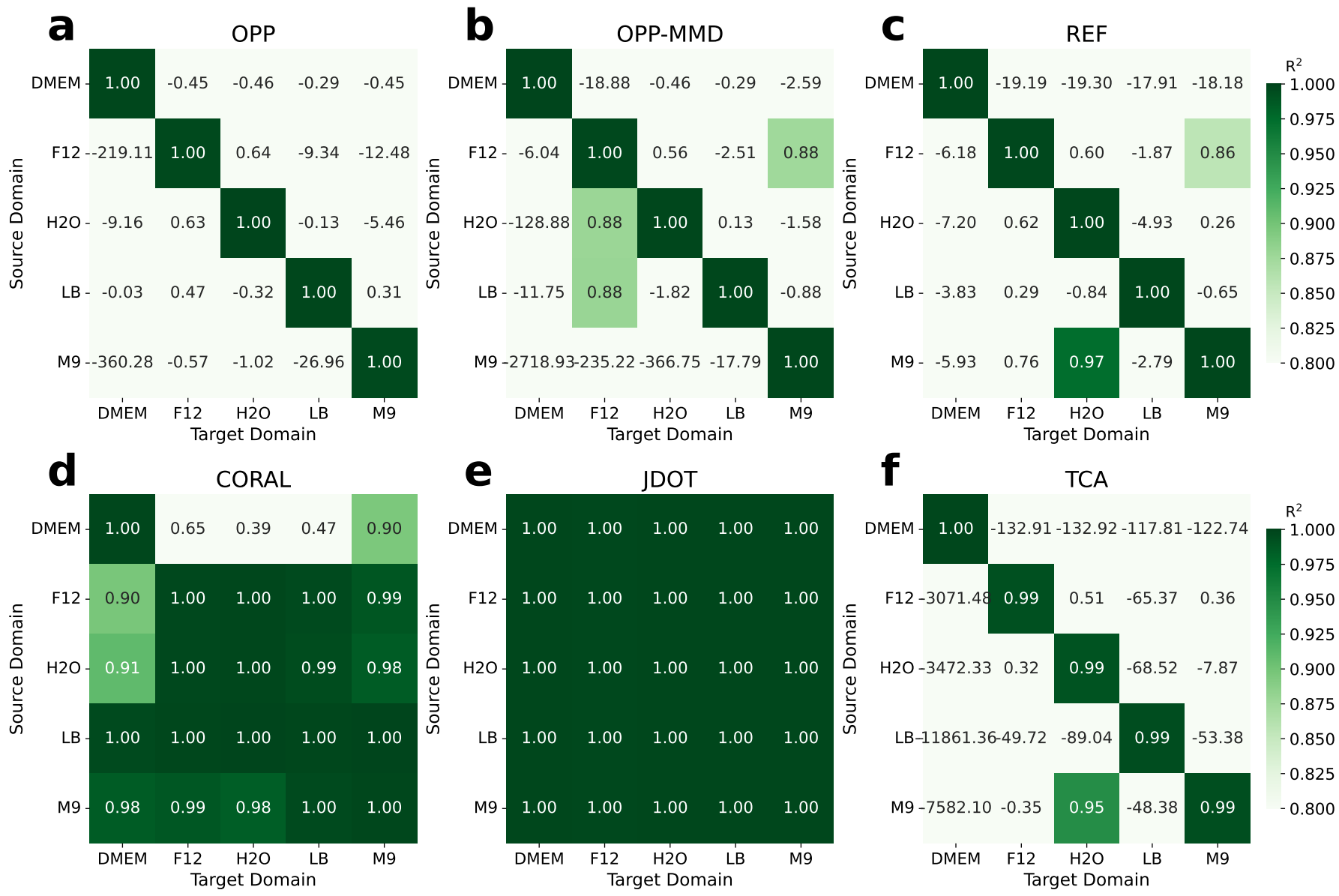


**Fig. S3**: Coefficient of determination ($R^2$) in Dataset A for six different modeling techniques upon introduction of a moderate peak shift. Each sub-plot corresponds to one model, showing performance when trained on one domain and tested on all others. Higher $R^2$ values indicate better agreement between predictions and true values. a) OPP = Optimal preprocessing, b) OPP-MMD = Optimal preprocessing chosen by maximum mean discrepancy, c) REF = Reference preprocessing, d) CORAL = Correlation alignment, e) JDOT = Joint distribution optimal transport, f) TCA = Transfer component analysis; each model workflow contains a preprocessing and/or transfer step followed by a PLS regression.

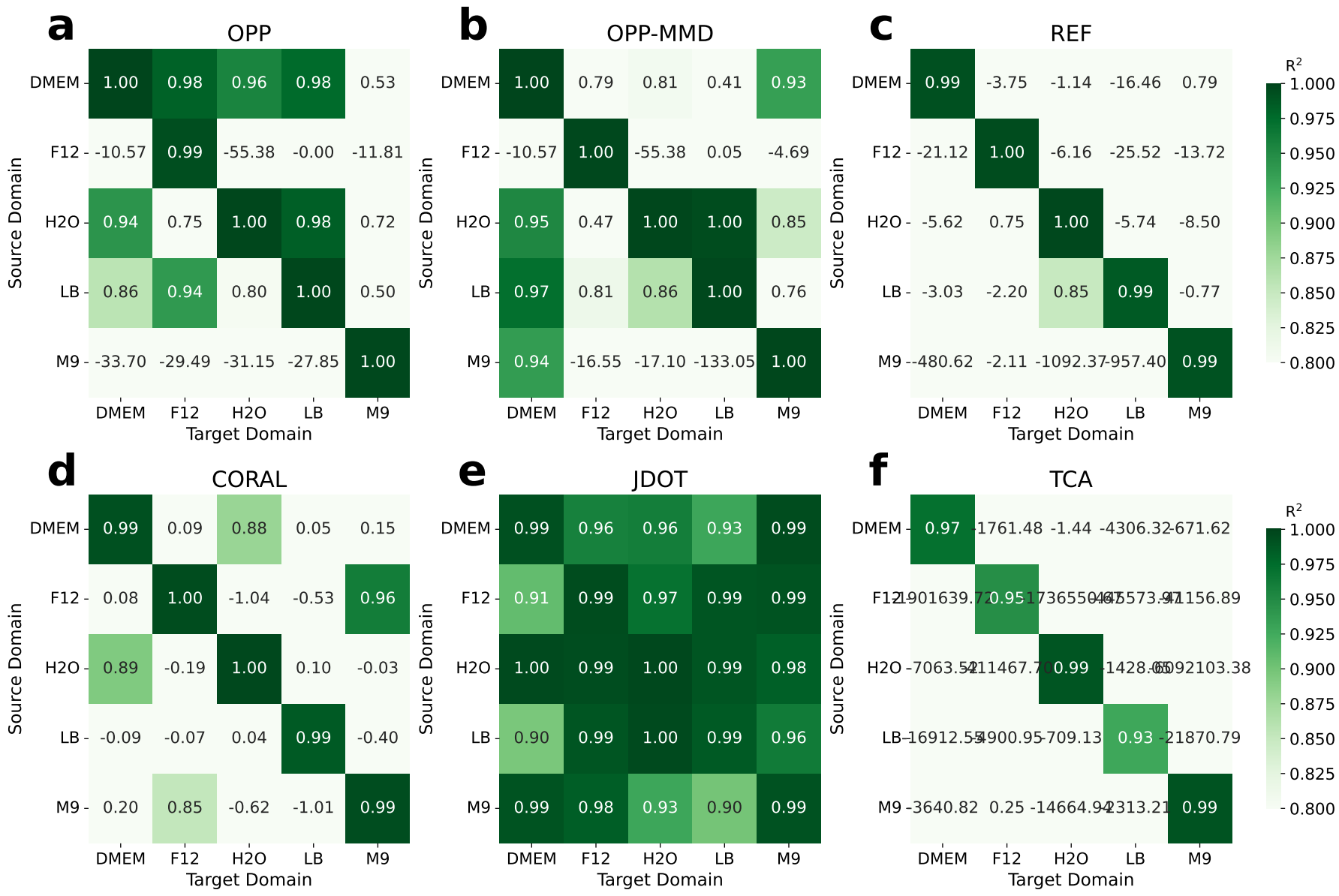

**Fig. S4**: Coefficient of determination ($R^2$) in Dataset A for six different modeling techniques upon addition of baseline shifts of varying intensity within a domain and varying type of shift between domains. Each sub-plot corresponds to one model, showing performance when trained on one domain and tested on all others. Higher $R^2$ values indicate better agreement between predictions and true values. a) OPP = Optimal preprocessing, b) OPP-MMD = Optimal preprocessing chosen by maximum mean discrepancy, c) REF = Reference preprocessing, d) CORAL = Correlation alignment, e) JDOT = Joint distribution optimal transport, f) TCA = Transfer component analysis; each model workflow contains a preprocessing and/or transfer step followed by a PLS regression.

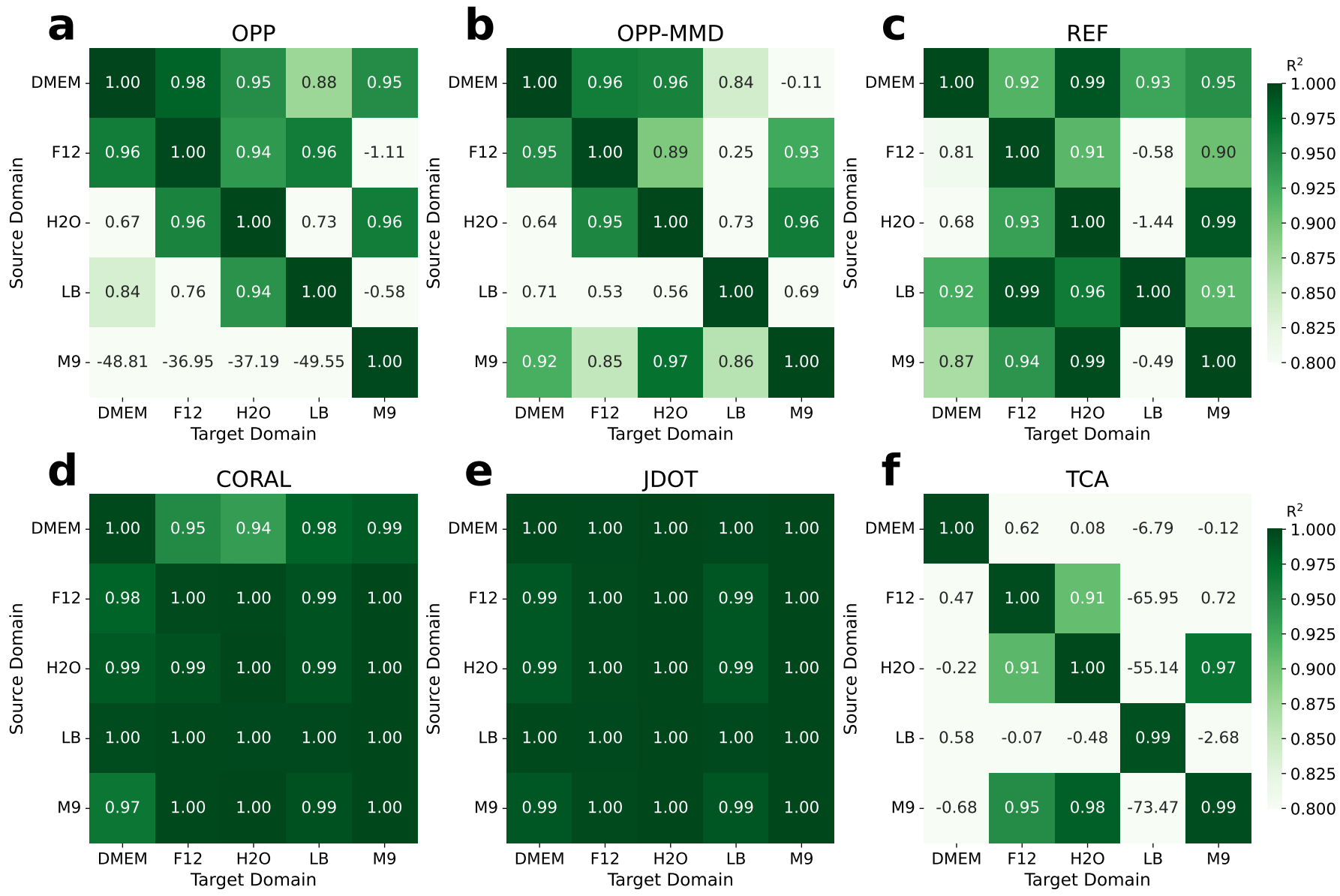


**Fig. S5**: Coefficient of determination ($R^2$) in Dataset A for six different modeling techniques upon scattering artifacts. Each sub-plot corresponds to one model, showing performance when trained on one domain and tested on all others. Higher $R^2$ values indicate better agreement between predictions and true values. OPP = Optimal preprocessing, a) OPP = Optimal preprocessing, b) OPP-MMD = Optimal preprocessing chosen by maximum mean discrepancy, c) REF = Reference preprocessing, d) CORAL = Correlation alignment, e) JDOT = Joint distribution optimal transport, f) TCA = Transfer component analysis; each model workflow contains a preprocessing and/or transfer step followed by a PLS regression.

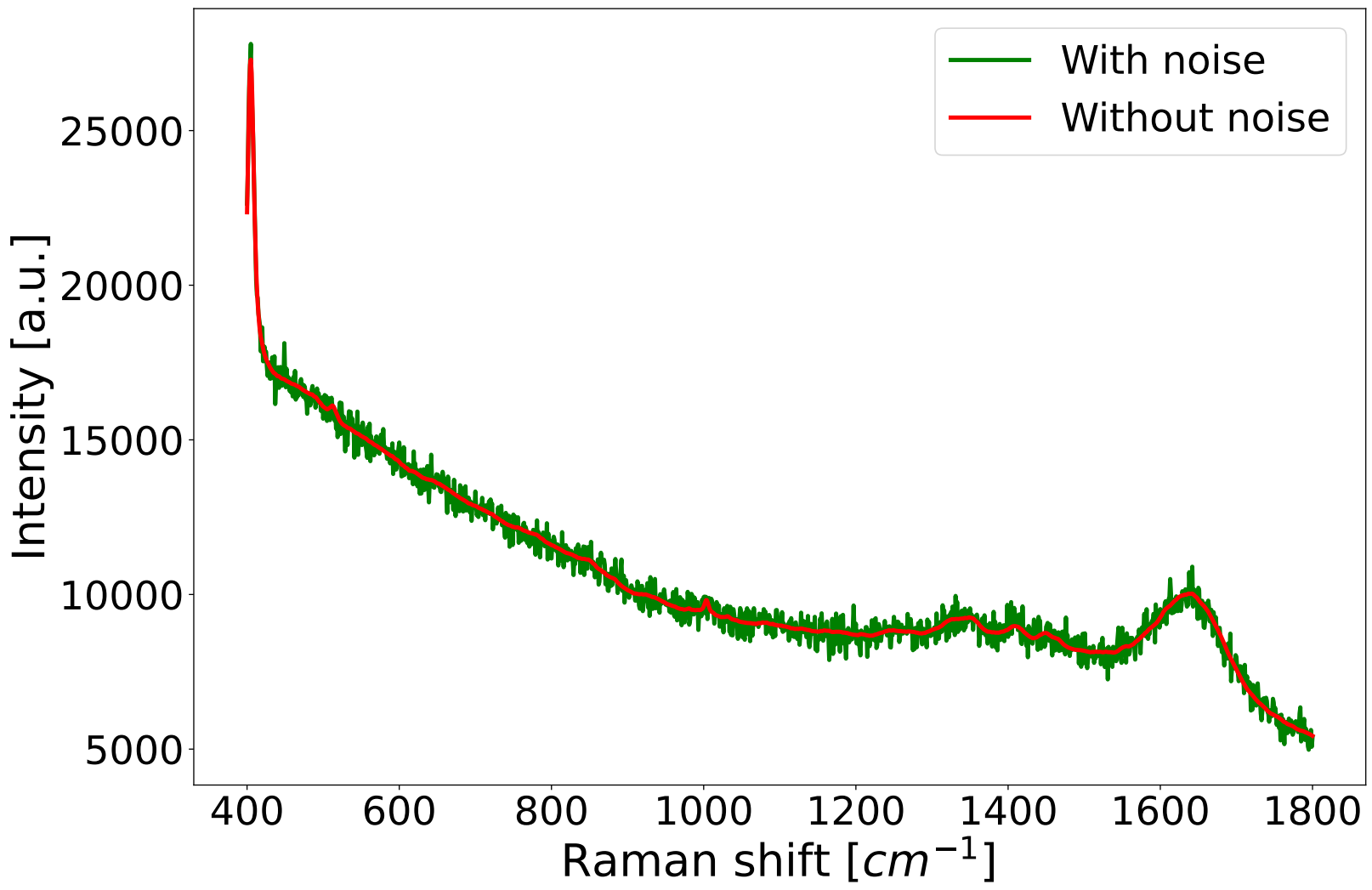


**Fig. S6**: Example of simulated white noise in Dataset A. Here, one spectrum of DMEM is shown as example (red line = original spectrum; green line = added white noise.)

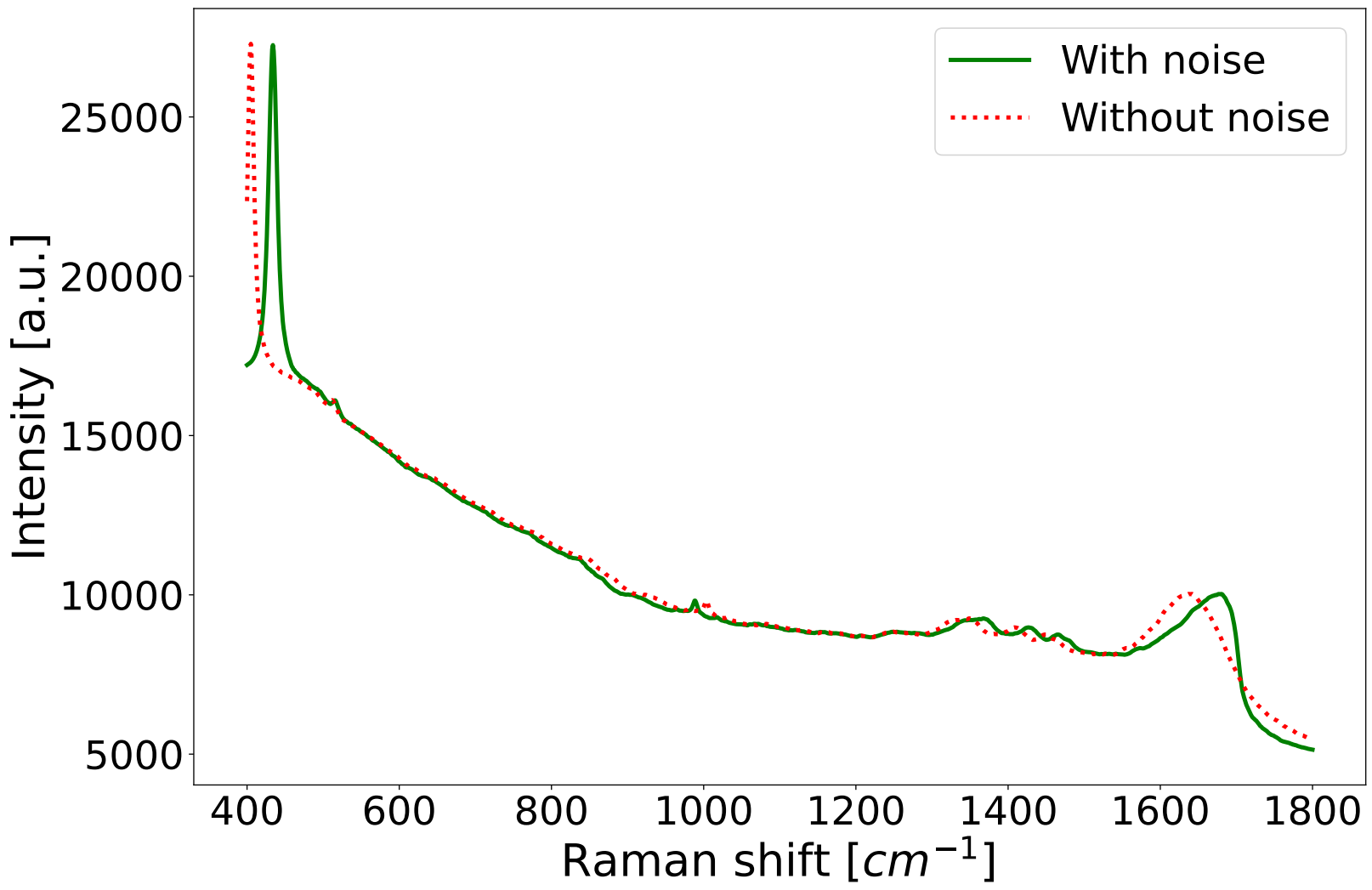


**Fig. S7**: Example of simulated peak shift in Dataset A. Peaks were varied between domains. Here, one spectrum of DMEM is shown as example (red line = original spectrum; green line = shifted peak.

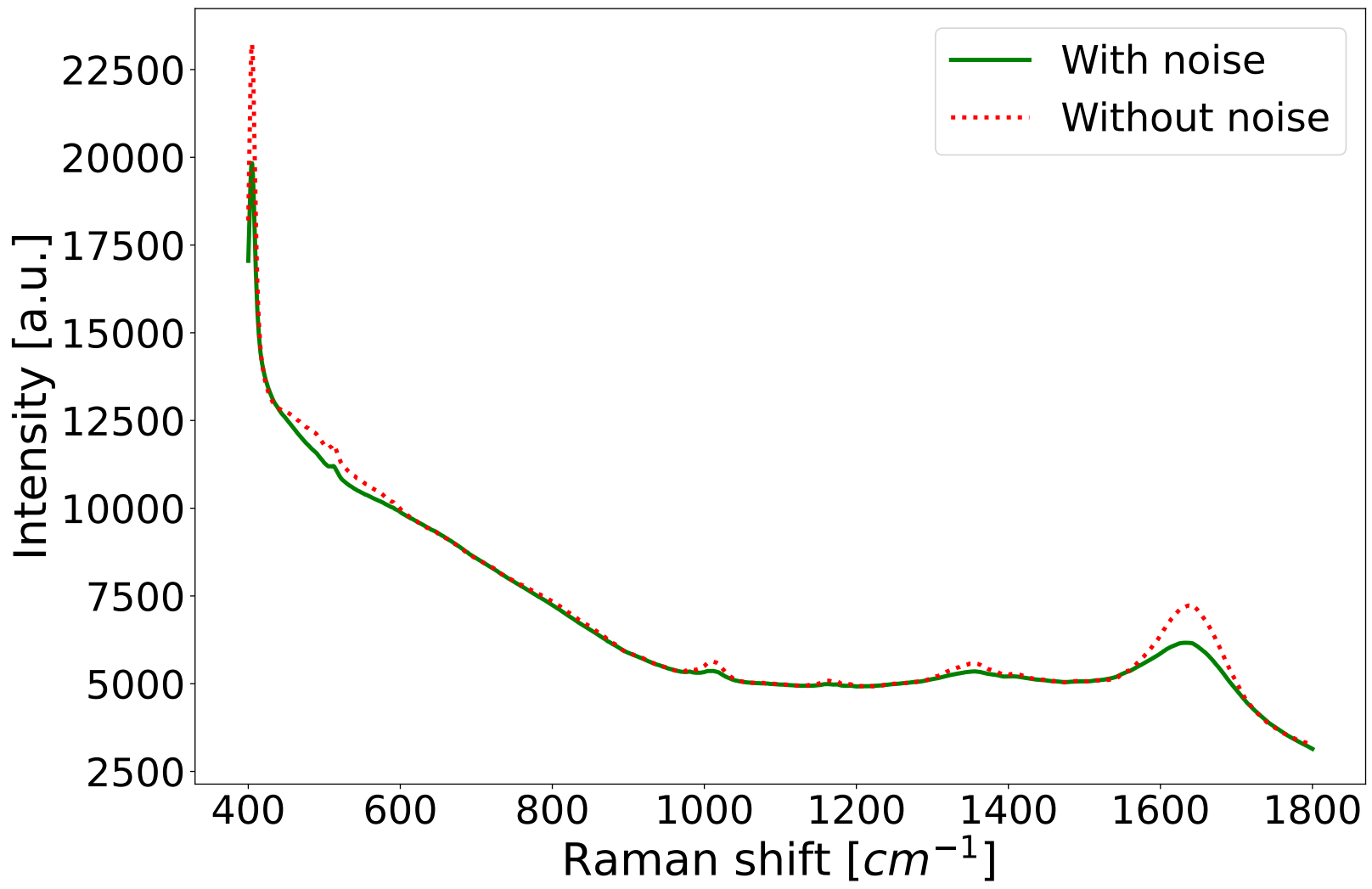


**Fig. S8**: Example of simulated scattering artifacts in Dataset A. Peaks were varied between domains. Here, one spectrum of DMEM is shown as example (red line = original spectrum; green line = attenuated signal.

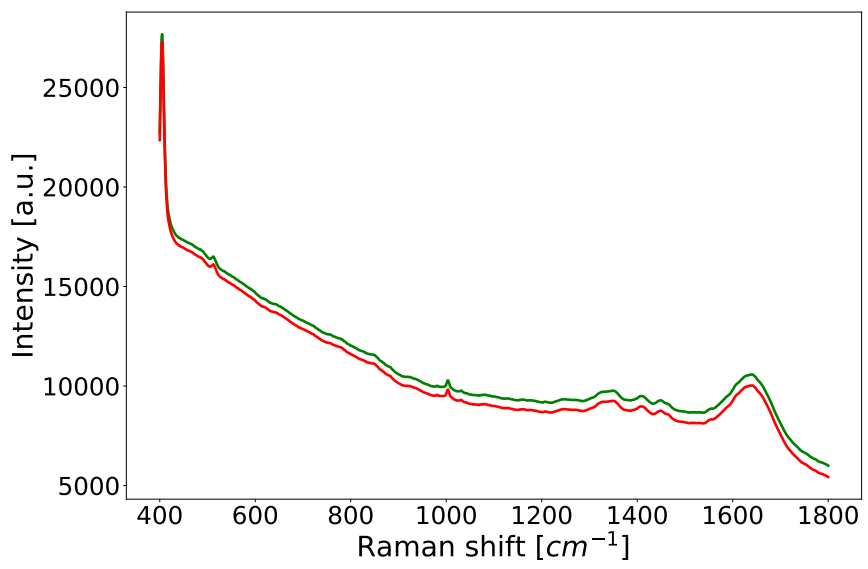


(a) Baseline drift DMEM

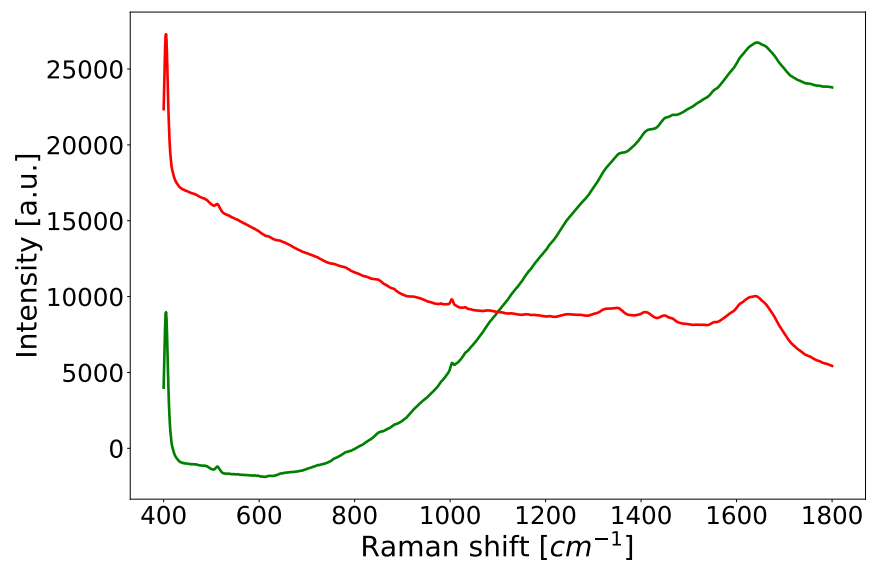


(b) Baseline drift F12

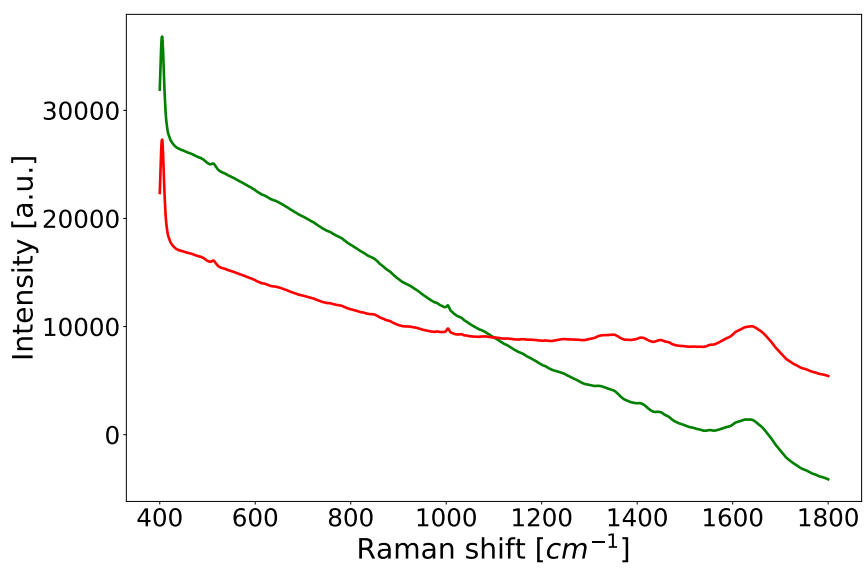


(c) Baseline drift H2O

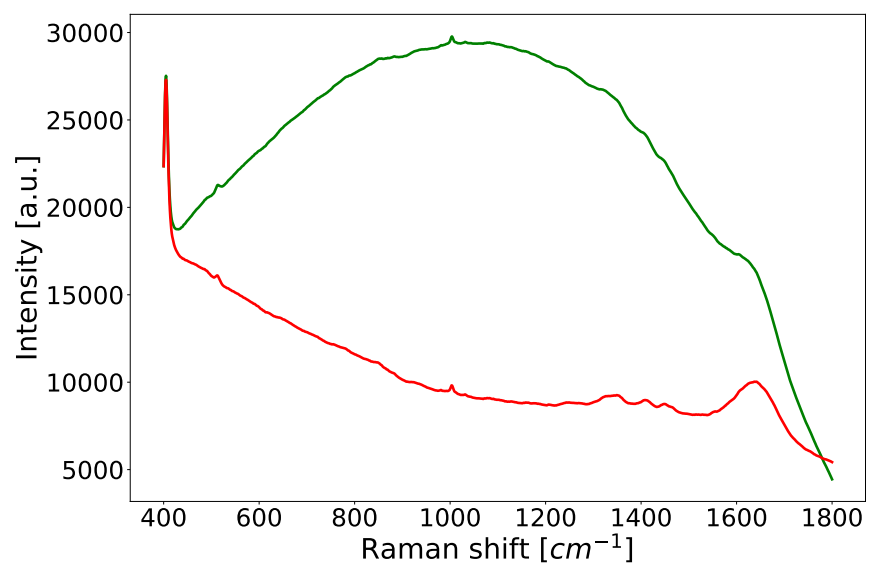


(d) Baseline drift LB

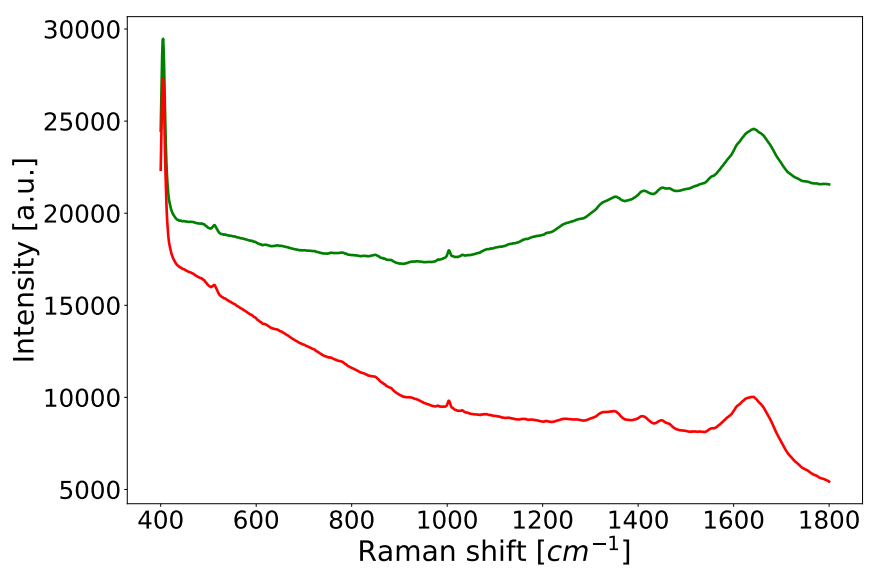


(e) Baseline drift M9

**Fig. S9**: Simulated baseline drifts in Dataset A. Within a medium, different intensities of the same drift were introduced (green). Original spectra are shown in red. Five spectra of each medium were plotted due to visibility in the plot. X-axis shows Raman Shift [$cm^{-1}$], y-axis intensity [a.u].

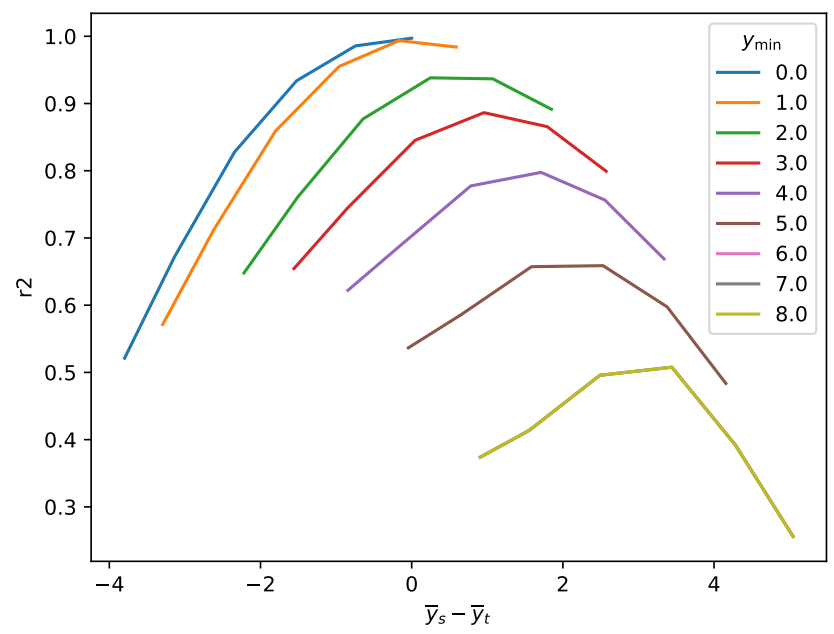


(a) $R^2$

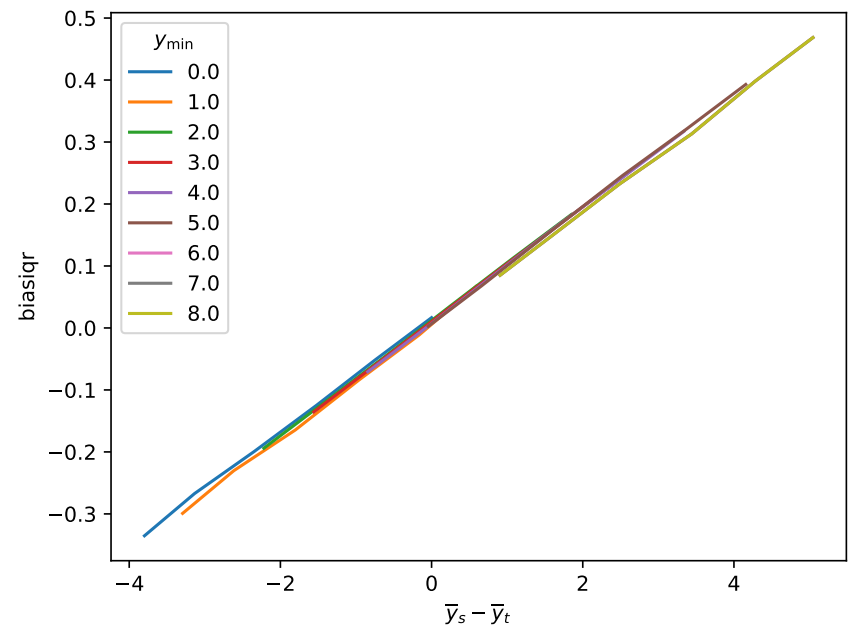


(b) Bias

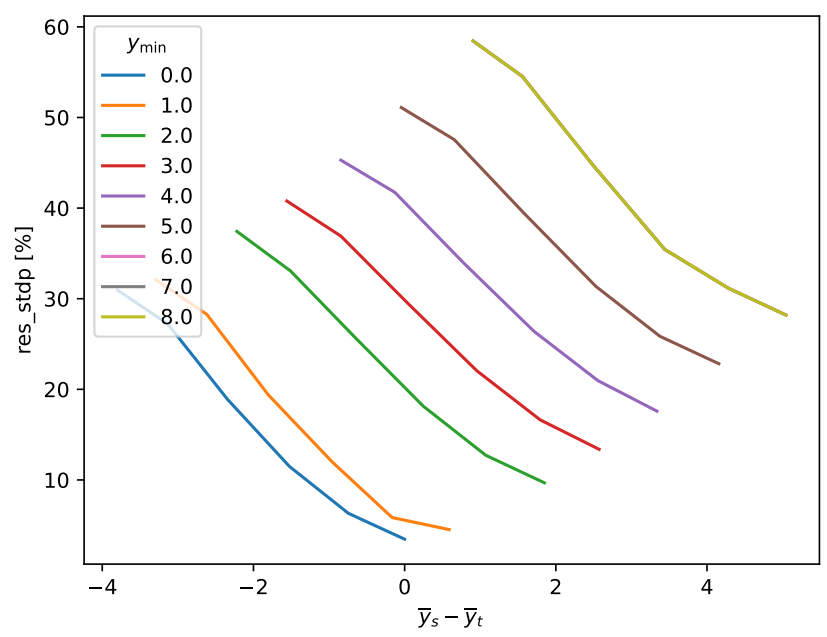


(c) Residual variance

**Fig. S10**: Comparison of impact of differing target and source data label distributions on JDOT performance. To simulate uneven source and target domain label distributions we truncated the source domain data from below and above so that only measurements between a minimum value $y_{\min}$ and $y_{\max}$ were considered. (a) The $R^2$ has a maximum if no truncation is done on the source domain data ($y_{\min} = 0$). The x-axis measures the difference between the average source domain label $\overline{y}_s$ and the average target domain label $\overline{y}_t$. Each line corresponds to data truncated by the value $y_{\min}$, while on each line the rightmost point corresponds to no truncation $y_{\max} = 20$ to maximum truncation $y_{\max} = 12$. The shapes of the lines show that clear maxima exist, which can be explained by considering the competing impact of the bias and residual variance. (b) The bias shows that all lines have minimum bias when the average source and target domain label coincides. (c) On the other hand the residual variance is minimal when the source and target domain variances are most closely aligned, which is always for the rightmost points of each line, when no truncation from above is done. These two effects explain, why we have a minimum to the right of 0 and shows that both variance and mean value of the label distributions play a crucial role for JDOT's performance.

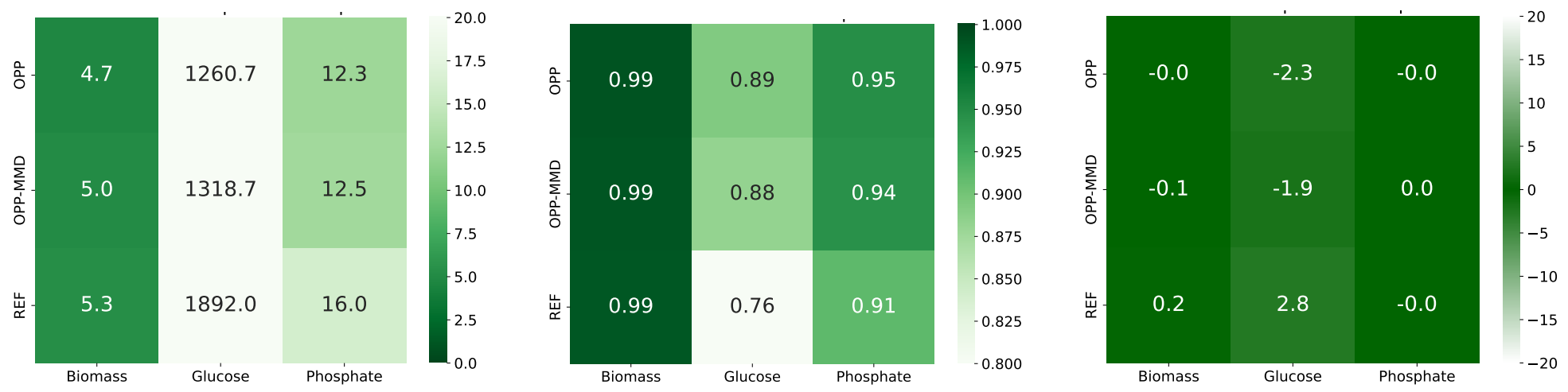


(a) RMSE [%] (b) $R^2$ (c) Bias [%]

**Fig. S11**: Performance metrics of source domain calculated by cross-validation (CV; 5-fold, random splits) on Dataset B.

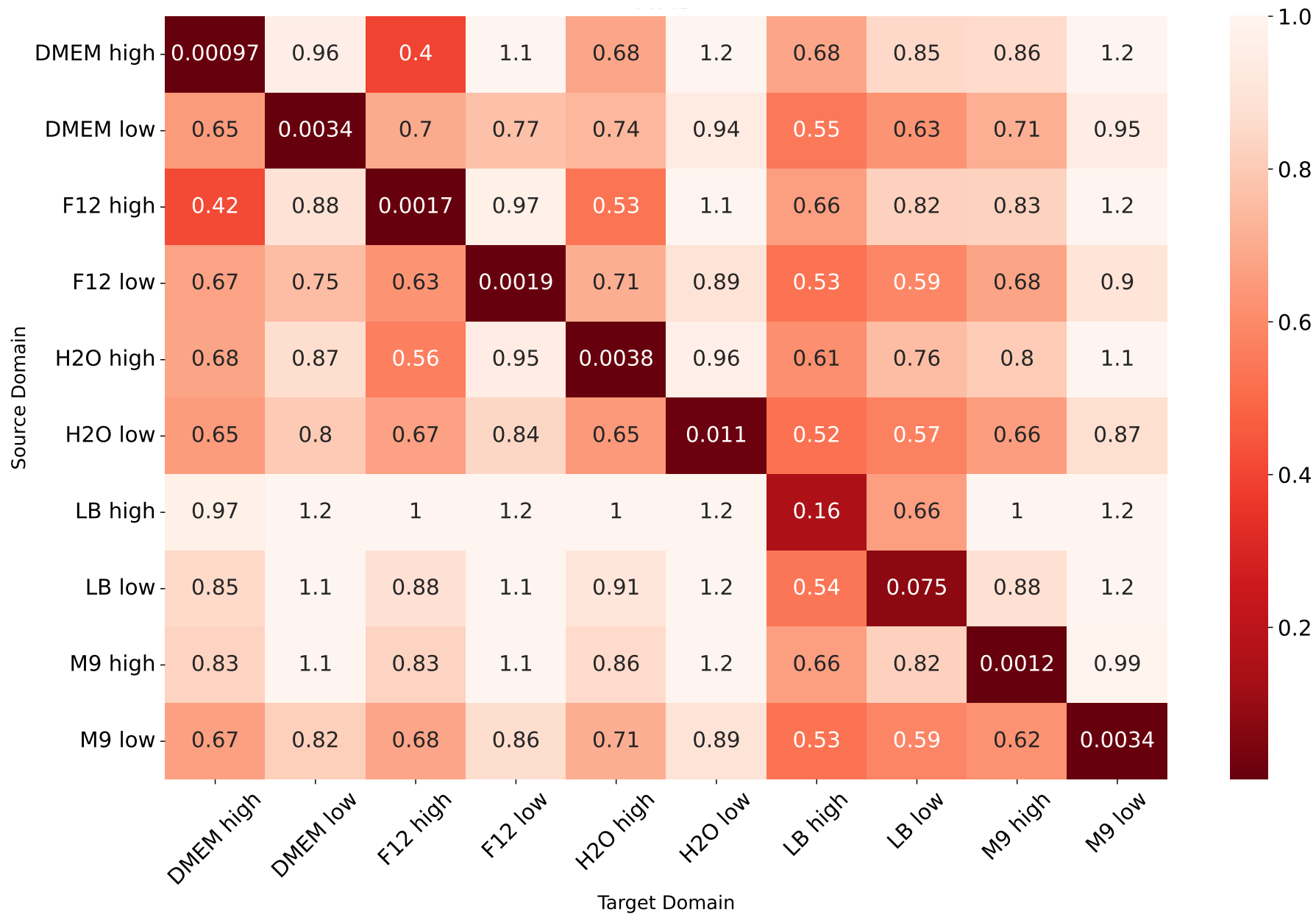


**Fig. S12**: Split-domain differences in Dataset A, quantified using the maximum mean discrepancy (MMD). Lower MMD values indicate greater similarity between the compared domains.

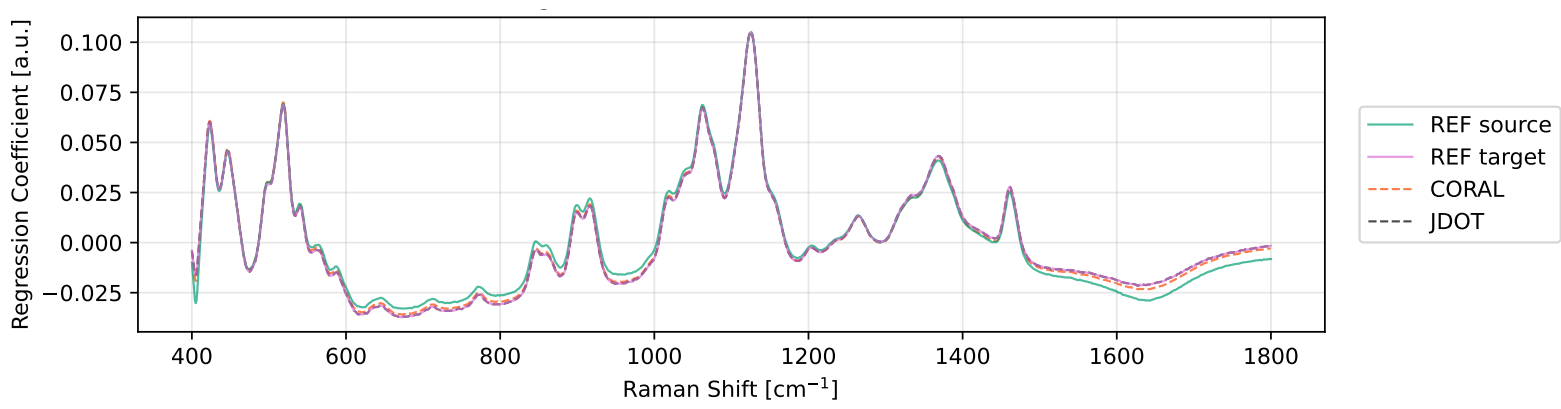


(a) Dataset A: Regression coefficient plot of glucose in water (REF source) and glucose in LB (REF target).

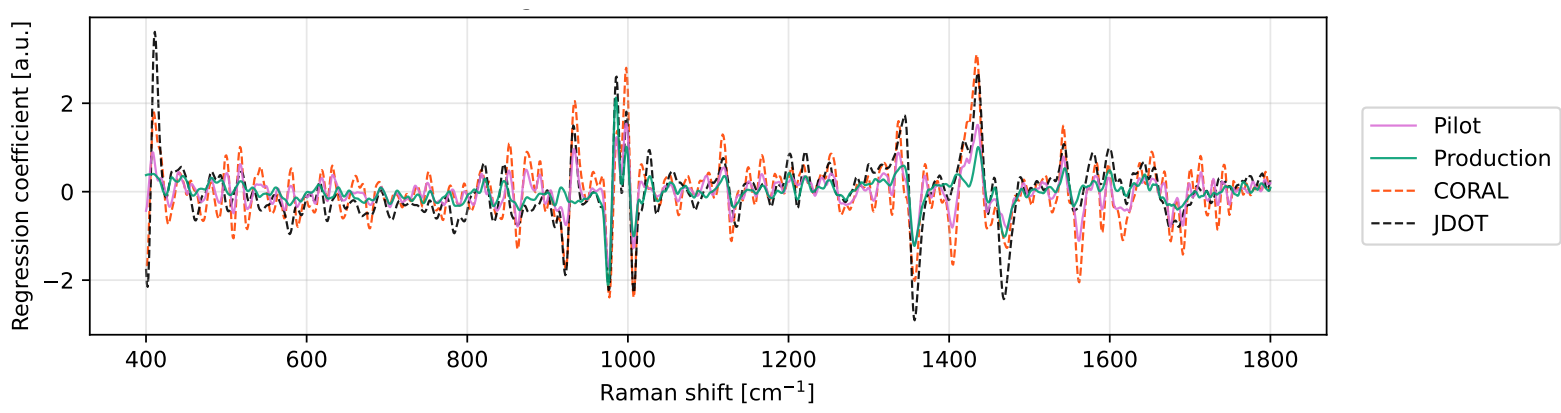


(b) Dataset B: Regression coefficients of biomass

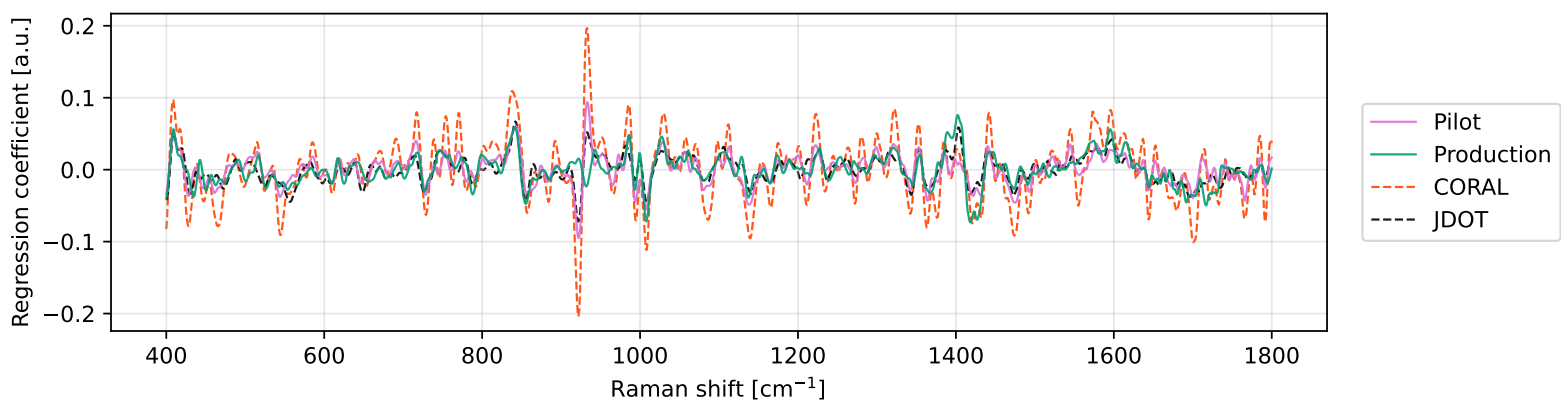


(c) Dataset B: Regression coefficients of glucose

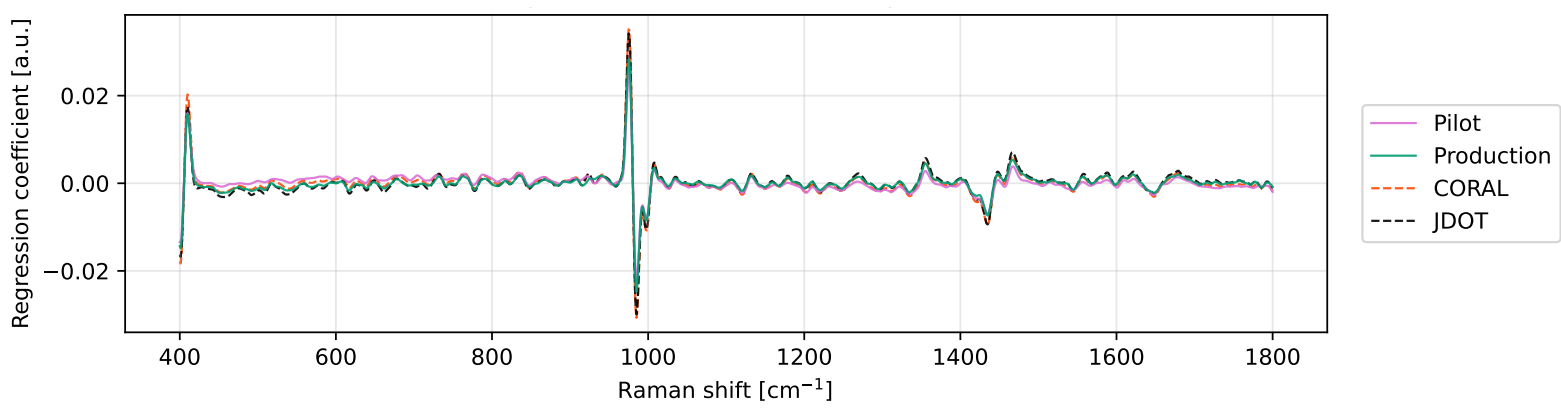


(d) Dataset B: Regression coefficients of phosphate

**Fig. S13**: Regression coefficients of analytes in Dataset A and Dataset B.

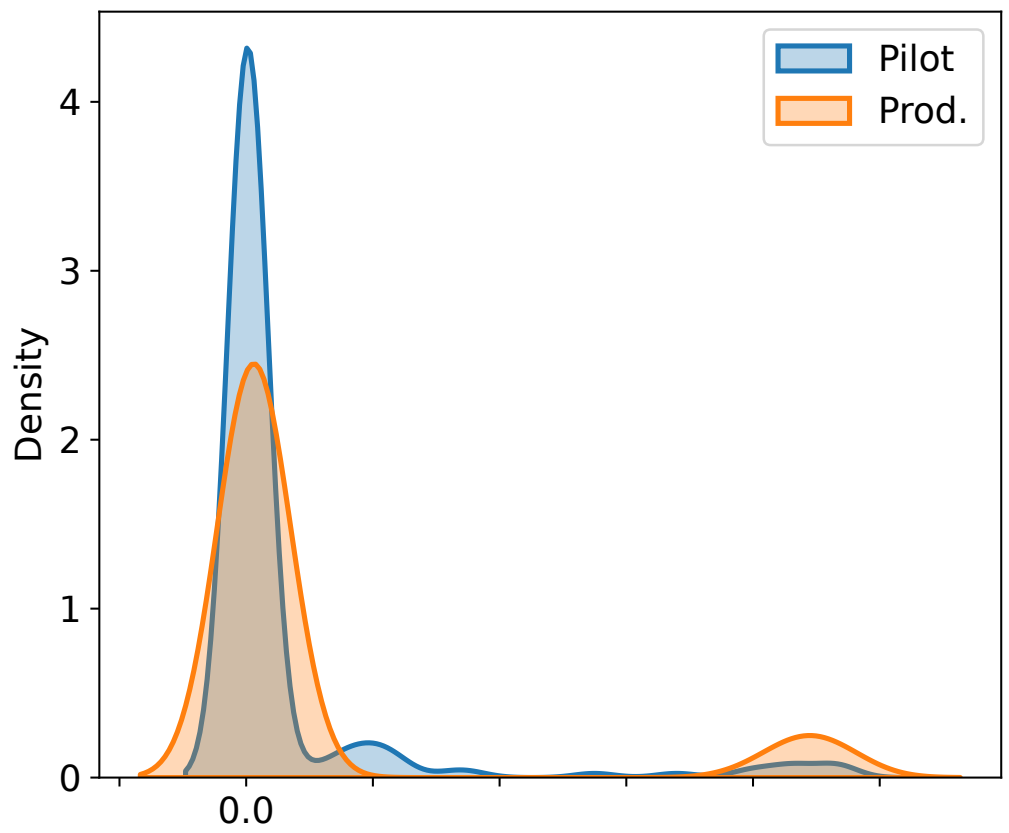


**Fig. S14**: Qualitative label distribution of glucose in pilot and production scale. The slight visual extension of the density estimate below zero is caused by kernel smoothing and does not correspond to negative glucose concentrations. Pilot and production scale each show a large peak/cluster of glucose values around zero and a smaller peak/cluster of elevated glucose values (Dataset B).
